# Supplementary material for: Emerging Protein Biomarkers for the Diagnosis or Prediction of Gestational Diabetes—A Scoping Review
Source: J Clin Med. 2021 Apr 6;10(7):1533. doi: 10.3390/jcm10071533 (PMC8038821; doi:10.3390/jcm10071533)
Supplement: Supplementary file 1 [file jcm-10-01533-s001.pdf]

Review

Emerging protein biomarkers for the diagnosis or prediction of gestational diabetes – a scoping review

Delia Bogdanet[1,2], Catriona Reddin[2], Dearbhla Murphy[2], Helen C. Doheny[2], Jose A. Halperin[3], Fidelma Dunne[1,2], Paula M. O'Shea[2],

[1] College of Medicine Nursing and Health Sciences, National University of Ireland Galway

[2] Galway University Hospital, Galway, Ireland

[3] Divisions of Haematology, Brigham & Women's Hospital, Boston, Massachusetts.

Delia Bogdanet; deliabogdanet@gmail.com

Catriona Reddin; reddin.catriona@gmail.com

Dearbhla Murphy; dearbhlaa.murphy@hse.ie

Helen Doheny; helen.doheny@hse.ie

Jose A. Halperin; jhalperin@bwh.harvard.edu

Fidelma Dunne; fidelma.dunne@nuigalway.ie

Paula M. O'Shea; paulaM.OShea@hse.ie ORCID 0000-0001-9392-1711

Corresponding Author

Delia Bogdanet

Email: deliabogdanet@gmail.com

Phone: 00353831027771

Address: Centre for Diabetes Endocrinology and Metabolism, Galway University Hospital, Newcastle Road, Galway, Ireland

ORCID 0000-0001-6127-5049

Abstract

Introduction

Gestational diabetes (GDM), defined as hyperglycaemia with onset or initial recognition during pregnancy, has a rising prevalence paralleling the rise in type 2 diabetes (T2DM) and obesity. GDM is associated with short-term and long-term consequences for both mother and child. Therefore, it is crucial we efficiently identify all cases and initiate early treatment, reducing foetal exposure to hyperglycaemia and reducing GDM-related adverse pregnancy outcomes. For this reason, GDM screening is recommended as part of routine pregnancy care. The current screening method, the Oral Glucose Tolerance Test (OGTT), is a lengthy cumbersome and inconvenient test with poor reproducibility. Newer biomarkers that do not necessitate a fasting sample are needed for the prompt diagnosis of GDM. The aim of this scoping review is to highlight and describe emerging protein biomarkers that fulfil these requirements for the diagnosis of GDM.

Materials and Methods

This scoping review was conducted according to Preferred Reporting Items for Systematic Reviews and Meta-Analyses (PRISMA) guidelines for scoping reviews using Cochrane Central Register of Controlled Trials (CENTRAL), the Cumulative Index to Nursing & Allied Health Literature (CINAHL), PubMed, Embase and Web of Science with a double screening and extraction process. The search included all articles published in the literature to July 2020.

Results

Of the 3519 original database citations identified, 385 were eligible for full-text review. Of these, 332 (86.2%) were included in the scoping review providing a total of 589 biomarkers studied in relation to GDM diagnosis. Given the high number of biomarkers identified, 3 post-hoc criteria were introduced to reduce the items set for discussion: we chose only protein biomarkers that had at least 5 citations in the articles identified by our search and published in the years 2017-2020. When applied, these criteria identified a total of 15 biomarkers which went forward for review and discussion.

Conclusions

This review details protein biomarkers that have been studied in an effort to find a suitable test for GDM diagnosis with the potential to replace the OGTT used in current GDM screening protocols. Ongoing research efforts will continue to identify more accurate and practical biomarkers to take GDM screening and diagnosis into the 21st century.

Keywords: gestational diabetes, biomarker, protein biomarker

Introduction

Gestational diabetes (GDM) is defined as hyperglycaemia with onset or initial recognition during pregnancy (1). GDM is a common complication of pregnancy, with a prevalence of 5.8- 12.9% globally, the prevalence varying by region and by diagnostic criteria (2). GDM is associated with substantial short and long-term adverse outcomes for both mother and child. Short-term complications include pre-eclampsia and pregnancy induced hypertension, increased risk of delivery by caesarean section, macrosomia, and neonatal hypoglycaemia (3, 4). Long-term complications include increased risk of type 2 diabetes mellitus (T2DM), obesity and cardiovascular complications for both mother and offspring (5, 6). Studies have established that effective treatment of GDM reduces the rate of short-term perinatal complications and improves the quality of life of the mother (7-9). Given this evidence, it is of utmost importance that we identify those at risk and accurately diagnose GDM (10). Current diagnostic strategies use the oral glucose tolerance test (OGTT) performed between 24 and 28 weeks of gestation, with universal screening advised in populations with high prevalence of T2DM (11).

As with any screening programme it is important that we continue to re-evaluate the test suitability, accuracy, and reproducibility. The OGTT was first described in 1957(12) and has been the gold standard for the diagnosis of GDM for decades(13). The OGTT is onerous, lengthy and requires a fasting state (14). A recent review by our research group(15) has detailed the numerous factors that contribute to its poor reproducibility (16).

In view of the cumbersome nature and poor reproducibility of the OGTT, it is necessary to look for and identify a more robust, convenient, and accurate biomarker for the diagnosis of GDM. Over recent years, substantial progress has been made in this field of biomarkers. There is an unmet clinical need to identify an easily measurable biomarker which is superior to the traditional OGTT. In addition, a more convenient biomarker could be used to diagnose GDM in early pregnancy reducing the period of intra-uterine hyperglycaemic exposure. This scoping review aims to synthesise the literature on emerging biomarkers for GDM diagnosis.

Materials and Methods

Scoping review question

What are the emerging biomarkers reported in the literature for the diagnosis of gestational diabetes?

Aim

The aim of this scoping review was to systematically identify the evidence available on emerging biomarkers with a potential to diagnose GDM (beyond glucose, fructosamine and HbA1c).

Methods

This review was conducted based on the framework for scoping reviews recommended by Arksey and O'Malley (17) and the later improvements to this method (18, 19). By contrast to systematic reviews, this approach was found to be more appropriate for a comprehensive search reflecting the vast number of biomarkers with a potential to diagnose GDM at the same time, enabling us to provide an in-depth analysis of selected key biomarkers(20). Scoping reviews are a method for recording evidence from a particular research area by presenting existing research results and highlighting gaps in the evidence at the same time.

Preferred Reporting Items for Systematic Reviews and Meta-Analyses (PRISMA) guidelines were followed using the PRISMA extension for scoping reviews checklist (21).

No review protocol for this study has been published.

Data Sources and Search Strategy

Using a broad-based search strategy, the following databases were searched for relevant studies from database inception through July 2020: Cochrane Central Register of Controlled Trials (CENTRAL), the Cumulative Index to Nursing & Allied Health Literature (CINAHL), PubMed, Embase and Web of Science. Search terms used included 'gestational diabetes', 'GDM', 'emerging/novel/new', 'biomarkers', 'tests', and 'diagnoses combined as appropriate using the Boolean operators 'AND' and 'OR' (Supplemental material).

Results were inputted into the reference manager, Rayyan web application (22), and duplicates were identified and removed. Two reviewers (DB and CR) screened the titles and the abstracts. The reference lists of included studies were also reviewed. Full texts of the remaining articles were independently assessed by two reviewers (DB and CR) for eligibility based on pre-defined criteria. Disagreements were resolved by consensus. Where a resolution was not reached by discussion, two other reviewers were consulted (FD, POS). The electronic search strategy can be found in (Supplemental Material)

Eligibility Criteria

Studies were eligible for inclusion if study participants were pregnant women, and the study reported on a biomarker for GDM diagnosis. All study designs were eligible for inclusion. We did not apply a language restriction, however if translation to English was not possible the study was excluded. There was no time restriction on the date of publication of the studies. Only full text articles were included in this review. When the full text was unavailable, the corresponding authors were contacted.

Data extraction and synthesis

Data was extracted independently by two authors (DB and CR) using a standardized pre-determined data collection form. For each study, we extracted the title, year of publication, journal, and biomarker (which was identified on review of the methods and results section of each paper).

Extracted data was compared for inconsistencies and merged into a final database. Disagreement was resolved through discussion and, where necessary, consultation with two further reviewers (FD, POS).

The biomarkers identified were grouped alphabetically together with all the papers citing the specific biomarker for easier identification.

It was decided that if the number of potential biomarkers identified was considerable rendering the analysis and discussion impractical, post-hoc criteria would be implemented. This would help focus the discussion on the most recent, most cited protein biomarkers.

Post-hoc inclusion criteria

Once all the biomarkers were identified, we selected for analysis and discussion biomarkers that fulfilled 3 criteria:

1. Protein biomarkers
2. Biomarkers that had at least 5 citations in our search results
3. Study publication year: 2017-2020

The resulting biomarkers were grouped into categories and brought forward for discussion.

Results

A total of 3519 articles were identified after the database search (Fig. 1). Following title screening and deletion of duplicates, 843 abstracts were selected. A total of 458 articles were further excluded after abstract screening by two researchers, thereby reducing the articles eligible for full-paper screening to 385. A total of 53 articles were excluded (articles not in English n=5, the test assessed was not used for GDM diagnosis n=13, no biomarker was discussed n=4, duplicates n=8 and conference proceeding/abstract publication only n=23). Finally, 332 articles were selected for data extraction. Following data extraction, a total of 589 biomarkers were identified (Supplemental Table 1).

After the application of the post-hoc criteria, 15 biomarkers were identified, reviewed, and discussed. These biomarkers were grouped into 3 categories: cytokines, glycoproteins, and other proteins (Table 1). The biomarkers' testing performance at the time of GDM and as a predictive indicator of GDM has been summarized in Table 2, Table 3and Supplemental Material Fig. 1 and Fig. 2.

1. Cytokines

Cytokines are cell signalling proteins, peptides or glycoproteins that are secreted by specific cells of the immune system. They regulate and modulate both the innate and adaptive immune response to inflammation and infection.

Adipokines

The adipokines are cytokines secreted by the adipose tissue and comprise a group of over 600 molecules that have paracrine and endocrine functions (23). Inflammation and dysfunction of the adipose tissue leads to a pattern of adipokines secretion which reflects a proinflammatory, dysmetabolic and diabetogenic model (23, 24).

a) Adiponectin

Adiponectin is a protein secreted primarily by the fat tissue but also by the brain, the skeletal muscle, and the placenta (25-27) comprising 244 amino acids. Adiponectin has a role in insulin sensitivity (28, 29), reduces liver gluconeogenesis (30) and enhances skeletal muscle fatty acid oxidation (31). Low adiponectin levels are associated with an increased incidence of T2DM (32, 33) and furthermore, low adiponectin levels were found in women with GDM (34, 35). This raised the question if adiponectin can be used to diagnose GDM.

Hedderson *et al*. (36) looked at the relationship of pre-pregnancy adiponectin levels and the risk of subsequent development of GDM in a case-control study within a cohort of 4098 women (GDM women n= 256, 100g 3-h OGTT, American College of Obstetricians and Gynaecologists criteria (37) controls n= 497). The team found that low adiponectin levels measured as far as 6 years prior to pregnancy were associated with increased risk of developing GDM independent of age, BMI, family history or ethnicity. This finding suggests that adiponectin could have the potential to identify women at high-risk of developing GDM who otherwise would not be classified as high risk. This study, however, does not capture the changes in lifestyle, diet, and exercise between the baseline adiponectin measurement and the GDM diagnosis and it also does not provide any information on body composition such as percentage of fat or anthropomorphic measurements. One study has found that first trimester of pregnancy adiponectin is significantly lower in GDM cases compared to controls and has the potential to determine the risk of developing GDM (38) with an AUC of 0.86 thus showing promise despite the small sample size of their cohorts (n=28). Similar results come from Williams *et al.* (39) and Ferreira *et al.* (40) who found that adiponectin levels taken at 13 weeks of gestation were lower in women who developed GDM compared to controls. Choosing a cut-off point of 9.1 μg/mL for first trimester adiponectin levels, Madhu *et al.* (41) found the test to have a sensitivity of 100% and a specificity of 95.6% in predicting GDM.

In 2018 Bozkurt *et al.* (42) investigated the relationship between adiponectin levels and the development of GDM. The study included 223 participants who were assessed for their glycaemic status (75g 2-h OGTT, IADPSG criteria) and adiponectin level at the first visit (<21 weeks of gestation) and at the second visit (24-28 weeks of gestation). The team found that adiponectin levels were significantly lower in women that developed GDM and the association between adiponectin levels and

GDM was even stronger in study participants that developed early GDM (<21 weeks), with a calculated predictive value for GDM of 0.67 (95% CI 0.57 to 0.77). Adiponectin taken during the OGTT at 24-28 weeks of gestation could predict GDM with an AUC of 0.65 (95% CI 0.57-0.74). These findings were independent of the pre-pregnancy maternal BMI; this is consistent with previous studies (43, 44) that found adiponectin levels to be similar between individuals with a normal BMI and obese individuals that are classified as being metabolically healthy (based on lipid levels, glycaemic status and blood pressure readings) compared to obese individuals classified as metabolically unhealthy. Therefore, in pregnancy, low adiponectin levels might indicate a pre-pregnancy predisposition for metabolic complications such as diabetes, hypertension, or dyslipidaemia rather than a reflection on the individual's adipose tissue mass. Weerakiet *et al.* (45) measured adiponectin levels in 359 women at the same time as the glucose challenge test between 21st and 27th week of gestation and while the results were consistent with previous findings in that adiponectin level are lower in women that develop GDM independent of age and BMI, in terms of screening however the AUC of adiponectin was less than the glucose challenge test (GCT) AUC (0.63 (95% CI 0.53-0.67) Vs. 0.73 (95% CI 0.71-080) and had a sensitivity of 91.7% and a specificity of 30.8%. These calculations, however, were based on arbitrarily chosen cut-off value for adiponectin at 10 µg/mL.

Xu *et al.* (46), in their systematic review and meta-analysis, looked at the association between adiponectin and GDM and included 15 studies and 560 GDM patients. They found that adiponectin levels were significantly decreased in women who developed GDM compared to controls, independent of BMI, similar to previous studies. The study however, had its limitations including a large variability in adiponectin cut-off points, and a high degree of heterogeneity. Iliodromiti *et al.* (47) conducted a systematic review and meta-analysis on the accuracy of adiponectin in predicting GDM and included 11 studies and data on 794 GDM women. They found that pooled sensitivity for adiponectin as a GDM diagnostic biomarker was 64.7 % (95% CI 51%, 76.4%) and the pooled specificity was 77.8% (95% CI 66.4%, 86.1% with an AUC of 0.78 (95% CI 0.74, 0.81). While the researchers conclude that adiponectin has a moderate predictive value there are several limitations to their paper including the study heterogeneity, the limited access to data (2 studies), the variability of adiponectin levels cut-off points for "low" or "high" levels, the diversity of ethnicities in the populations involved and the various study designs and retrospective nature of the data that might have contributed to the results.

Adiponectin is a very promising biomarker for the diagnosis of GDM and has a significant advantage over the OGTT/GCT of not mandating a fasting state for measurements (48). While some studies determined less than ideal performance parameters for adiponectin, we need to consider that in the first trimester fasting glucose has been shown to have a sensitivity of 47%, a specificity of 77% and AUC of 0.62 (49) improving in the second trimester (50); HbA_{1c} has a sensitivity of 32% and a specificity of 94% (51) and fructosamine has a sensitivity of 12.2% and a specificity of 94.7% (52). More so, there are no studies assessing adiponectin level cut-off points for best prognostic/diagnostic capacity nor are any studies on adiponectin trimester specific interval ranges.

Large prospective studies together with health economic input analysing best diagnostic cut-off points, natural level variation in GDM and normal glucose tolerance (NGT) cohorts, the impact of confounders such as ethnicity, percentage of body fat etc are required to accurately determine the true value of adiponectin in diagnosing GDM.

b) Chemerin

Discovered more than 20 years ago (53), chemerin (163 amino acids) is an inflammatory adipokine with a role in adipogenesis, adipocyte metabolism (54) and insulin resistance (55) secreted from the adipose tissue, liver, intestine (56) and placenta (57). Chemerin plays a role in adipocyte metabolism, inflammation, insulin resistance and metabolic processes (54, 55, 58) and chemerin levels have been associated with adverse pregnancy outcomes (59-61).

Yang *et al.* (62) measured chemerin levels in the first trimester of pregnancy (8-12 weeks' gestation) in 212 women and in 39 women (GDM n=19, IADPSG criteria) after the 75g 2-h OGTT. Chemerin levels were significantly lower in the GDM group compared to NGT in the first trimester but significantly higher in the third trimester. In both GDM and NGT groups, chemerin significantly rose between the first and 3rd trimester paralleling the rise in HOMA -IR.

In 2020 Wang *et al.* (63) found that the AUC of chemerin (cut-off value 6.78 µg/L) in the diagnosis of GDM (24-28 weeks of gestation) was 0.82 (95%CI 0.74-0.89) with a sensitivity of 73.3% and specificity of 76%.

Pfau *et al.* (64) measured chemerin levels in 40 GDM women and, while the levels were higher in GDM subjects, there was no significant difference when compared to controls; there was however an independent association between chemerin and markers of insulin resistance . Guelfi *et al.* (65) measured adipokine levels including chemerin in 123 pregnant women at 14 and 28 weeks of gestation and found no change in chemerin concentration between the 2 time points and no difference in chemerin levels between women who developed GDM compared to those who did not (unlike adiponectin and leptin that showed significant changes). The cohort in this study included only women with a history of GDM with a different metabolic profile compared to the general population so the results cannot be extrapolated. Van Poppel *et al.* (66) found no difference in chemerin levels between GDM (n=15, IADPSG criteria) and NGT subjects. However, chemerin levels were significantly higher in obese women compared to non-obese women. In their systematic review and meta-analysis (10 studies), Sun *et al.* (67) did not find any difference in chemerin levels between GDM and NGT women but did find a positive correlation between chemerin and BMI. These results contradict the findings of the Zhou *el al.* (68) who conducted a systematic review and meta-analysis (11 studies) looking specifically at chemerin levels and GDM and found that chemerin levels are significantly raised in the GDM population compared to NGT women. While both systematic reviews and meta-analyses had significant heterogeneity, the discrepancy in results might arise either from the different studies comprising the analysis or either from the type of sub-analysis and confounders included.

The main reason for the discordant results is the fact that chemerin is influenced by numerous factors such as inflammation, insulin resistance, metabolic syndrome, obesity, diabetes, nutrition, activity level and pregnancy (57, 59, 69-73). It seems that chemerin might play a better role as a risk-stratifying tool rather than a GDM diagnostic biomarker, identifying women at risk of GDM but future research might prove otherwise.

c) Fetuin

Fetuins are a group of adipokines mainly secreted by the liver. Fetuin A is secreted from the liver and adipose tissue with elevated levels in obesity (74, 75), metabolic syndrome (76), fatty liver disease (77), and T2DM (78, 79). Fetuin B, secreted by hepatocytes, tongue and placenta (80), is increased in hepatic steatosis and is linked to gluconeogenesis through insulin suppression (81, 82). Based on the association with insulin resistance and glucose metabolism, it was hypothesized that fetuins can serve as markers for GDM diagnosis.

Kansu-Celik *et al.* (83) measured first trimester fetuin-A as a biomarker for GDM diagnosis in 88 pregnant women (GDM n=29, GCT/OGTT, Carpenter and Coustan criteria) and found significantly lower levels in GDM women compared with controls. Fetuin-A below 166ng/mL could predict GDM with a sensitivity of 58.6%, specificity of 76.2% and AUC of 0.337 (95% CI 0.21-0.46).

Kalabay *et al.* (84) measured Fetuin-A in 134 pregnant women (GDM n=30, 75g 2-h OGTT, 1999 WHO criteria) and 30 non-pregnant women in each trimester of pregnancy (including at the time of the OGTT) and found significant higher levels of fetuin-A in GDM women at all time points compared to the NGT and non-pregnant women; fetuin A was also positively associated with markers of insulin resistance, TNF-α and leptin levels. Iydir *et al.* (85) also found higher fetuin-A levels (sample collected at the time of the OGTT) in GDM women (n=26, Carpenter and Coustan criteria (86)) compared to NGT and decreased post-partum. The authors found a positive correlation between fetuin-A and HbA1c levels. Jin *et al.* (87) measured fetuin-A in 270 women (GDM n=135, IADPSG criteria) in the first and second trimester of pregnancy and found significant higher levels of fetuin-A in GDM women compared to controls at both time points and it was positively correlated with the changes in the markers of insulin resistance. In this study, a fetuin-A cut-off value of 305.9pg/mL in the first trimester would predict GDM with a sensitivity of 64.4%, specificity of 58.5% and AUC of 0.61 (95% CI 0.54 to 0.68).

Farhan *et al.* (88) measured fetuin- A in 20 women (GDM n=10) at the time of the 75g 2-h OGTT (28 weeks of gestation) and 3 months post-partum; they found no difference in fetuin-A levels between GDM and NGT study participants at any time point.

The discrepancy between these study results arises from the different study designs, different population characteristics and sample size, and the different time-point sampling making the results inconsistent and difficult to compare.

It is unclear what the exact role of fetuin-A is in the pathophysiology of GDM. Some hypotheses suggest that its main action is through insulin resistance through the inhibition of the insulin receptor while others suggest that fetuin-A induces adipose tissue inflammation which leads to lipid-induced insulin resistance. There is even less information on fetuin-B, as its mode of action, signalling and even receptor have not been adequately described. However, the minimal studies available show promising results. Prospective studies, with longitudinal sampling of both fetuins while assessing correlations with markers of insulin resistance are required.

d) Leptin

Leptin (167 amino acids), the first adipokine to be discovered in 1994, (89) is predominantly secreted by adipose cells (90) but also by the stomach (91), placenta (92) and the brain (93). Leptin has a role

in energy homeostasis by inhibiting hunger and mediating food intake (94, 95) through its action on the hypothalamus, dopamine system and brain stem (96). More so, in both animal and human models, leptin administration improved hyperinsulinemia, hyperglycaemia, insulin resistance and hyperlipidaemia (97-99).

While leptin levels rise in pregnancy compared to the non-pregnant state, peaking between 20-30 weeks of gestation most likely secondary to fat accumulation (48, 100), even higher leptin levels have been associated with GDM.

Bawah *et al.* (101) found that first trimester leptin levels (11-13 weeks of gestation) in 140 women (GDM n=70, 75g 2-h OGTT, American Diabetes Association (ADA) criteria(102)) could predict the development of GDM with a sensitivity of 95.7%, specificity of 68.6% and AUC of 0.81.

Kautzky- Willer *et al.* (103) measured leptin levels at 28 weeks of gestation in GDM women (n=55, 1999 WHO criteria (104)), women with NGT (n=25) and women with T1DM (n=10). These samples were collected in a fasting state and 30 minutes after the glucose load during the OGTT. They found that leptin levels were higher in women with GDM compared to NGT and T1DM, and similar between NGT women and T1DM women, all matched for BMI. There were no differences between leptin levels between fasting and post-glucose load values indicating that this test could be done in a non-fasting state. Boyadzhieva *et al.* (105) measured fasting leptin levels during the OGTT in 286 women (GDM n= 127, IADPSG criteria) and found significant higher levels in the GDM group compared to the controls. They also assessed if leptin could be used as a screening test and, setting the cut-off value at 28.7 ng/mL, the test could exclude GDM with a sensitivity of 81.2%, a specificity of 64.2% and AUC of 0.827. Bozkurt *et al.* (42) found higher levels of leptin in women with GDM compared to controls but the predictive value for GDM was 0.66 (95% CI 0.57 to 0.74). Leptin taken during the OGTT at 24-28 weeks of gestation could identify GDM with an AUC of 0.61 (95%CI 0.53-0.69).

Contradictory results come from the work of McLachlan *et al.* (107) who found higher leptin levels in the control group compared to GDM. However, this was of borderline significance p=0.05. More so, the number of women in this study was small (19 women in each arm) but well matched and the measurements for leptin levels were taken during an intravenous glucose tolerance test (IVGTT) in the third trimester of pregnancy. While the OGTT is preferred over the IVGTT in detecting glucose intolerance (108), we also know from previous studies (48, 100) that leptin levels peak up to 30 weeks of gestation and start decreasing thereafter.

In a systematic review and meta-analysis Xu *et al.* (46) found that high levels of leptin in early pregnancy may be predictive for developing GDM independent of BMI. In their systematic review (which included 9 prospective studies), Bao *et al*. (106) found that leptin levels taken in the first or second trimester of pregnancy were 7.25 ng/ml higher (95% CI 3.27-11.22) in women who were subsequently diagnosed with GDM compared to women with NGT

The data on leptin is slightly contradictory and that might be due to the leptin correlation with adipose tissue. Despite that, most studies show great promise. While there is some evidence that stress, sleep deprivation or exercise influence leptin levels (109-111), similar to the OGTT (15), the test can be done in a non- fasting state which is a clear advantage over the OGTT. Similar to adiponectin, prospective studies are required to determine trimester specific reference ranges for non-diabetic pregnant population, trimester-specific cut-off points for the GDM population, the impact of confounders (including adiposity markers) on leptin levels and association with pregnancy outcomes.

e) Omentin

Omentin (313 amino acids) is an adipose-tissue specific factor selectively expressed in visceral tissue relative to subcutaneous adipose tissue. Omentin has a role in fat distribution, energy expenditure and insulin action modulation (112, 113). In 2007, de Souza Batista *et al*. (114) found that omentin levels correlated negatively with BMI/obesity, leptin level, and markers of insulin resistance and correlated positively with HDL and adiponectin levels in healthy subjects.

Barker *et al.* (115) studied the effects of pregnancy on omentin levels also assessing the impact of BMI and GDM on omentin levels. Blood samples were collected in the first and second trimester from 83 pregnant women (GDM n=39, 75g 2-h OGTT, Australasian Diabetes in Pregnancy Society (ADIPS) (116)). The study found significantly decreased omentin levels in non-obese GDM women compared to controls with no difference in levels between obese GDM and NGT study participants. Omentin was negatively associated with fasting glucose and maternal BMI and no association was found between omentin and adiponectin or leptin levels. While the subgroup numbers were small and the outcomes most likely underpowered, this research was one of the first to explore the role of omentin in pregnancy and GDM raising further questions such as what is the balance between omentin secretion and clearance at each stage of the pregnancy; does the ratio between adipose tissue omentin secretion and placental tissue omentin secretion change during pregnancy?

Abell *et al.* (117) measured omentin levels in the first trimester of pregnancy in 103 women (25 of whom later developed GDM) and found lower omentin -1 levels in women with GDM compared to controls and a negative association with 1-h and 2-h glucose levels of the OGTT. They also found that omentin-1 levels less than 38.36 ng/ml were associated with a 4-fold increased risk of GDM, and that for 1ng/ml increase in omentin levels the risk of GDM was OR 0.97 (95%CI 0.94-0.99). Limitations of the study include that all the participants in this study were high risk for GDM with all pregnant women being overweight or obese and the women were initially screened with a GCT followed by the OGTT if deemed necessary with arguably milder GDM cases being missed (thus this is only attributable to the very highest risk group and not suitable for population screening). Regardless, this study also highlights that trimester 1 omentin might have the potential to predict GDM.

Contradictory data comes from Franz *et al.* (118) who measured omentin levels in 192 pregnant women (GDM n=96, German and Austrian Society for Diabetes criteria based on the Hyperglycaemia and Adverse Pregnancy Outcomes (HAPO) study (119)) at the time of the OGTT, at 32 weeks and from the umbilical cord at the time of the delivery. While omentin levels were lower in the GDM group compared to the NGT group at all timepoints, this was only statistically significant at the delivery timepoint. Omentin levels were also lower in women with a higher BMI and a lower HDL cholesterol.

In a systematic review and meta-analysis which included 20 studies (GDM n= 1493), Sun *et al.* (67) found that omentin levels were significantly lower in women with GDM than in healthy controls. The authors also suggested that age and BMI might be important parameters influencing omentin levels in GDM patients. While there was significant heterogeneity in this review, and limited number of studies identified, the authors conclude that omentin has the potential to be a novel biomarker for early GDM diagnosis.

Omentin shows some promise as a GDM diagnostic biomarker, however further studies are required to clarify the actual role in GDM pathophysiology – if it is linked to visceral adiposity, vascular/endothelial dysfunction in either visceral adipose tissue or placenta or insulin mediation. Prospective studies are required to detect specific reference ranges and cut-offs and assess the impact of adiposity and inflammation on omentin levels and consecutively on omentin capacity to diagnose

GDM. More so, omentin levels are influenced by fasting state (120, 121) which makes it a less attractive biomarker compared to other biomarkers discussed.

f) Interleukin 6 (IL-6)

IL-6 is an inflammatory cytokine (122) secreted by monocytes/macrophages but also endothelial cells, myocytes, adipocytes, pancreatic cells and placenta (122, 123) with primary roles in immune response regulation, inflammation and haematopoiesis (124) but also roles in obesity, insulin resistance and T2DM (125-127). Some of the mechanisms proposed for its role in metabolism are the percentage of body fat (128), the degree of visceral fat (129), IL-6 direct effect on hepatocytes (125, 130), immune response induced dyslipidaemia, IL6 lipolytic effect (131) or even a central effect of IL-6 on food intake (132, 133). A systematic review and meta-analysis by Liu *et al.* (Liu 2016) explored the association between IL-6 and T2DM. It comprised 16 studies involving 24,929 subjects and found that IL-6 was a strong predictor of developing T2DM. In pregnancy, the role of IL-6 in GDM prediction has given conflicting results.

Sudharshana Murthy *et al.* (134) explored the role of IL-6 in GDM. IL-6 levels were taken at the time of the OGTT in 60 pregnant women (GDM n=30, OGTT) and found significantly raised IL-6 levels at the time of diagnosis. Siddiqui *et al.* (135), using a very similar study design, measured IL-6 levels in 103 pregnant women (GDM n=53, OGTT, ADA criteria) at the time of the OGTT and found significantly increased IL-6 levels in the GDM cohort compared to the NGT and a strong association between IL-6 levels and pre-pregnancy BMI and fasting and post-prandial glucose levels. The participants in both studies were Asian with a median normal/ normal- high BMI.A prospective study by Braga *et al.* (136) involving 176 South American pregnant women (GDM n=78, 100g OGTT, Carpenter and Coustan criteria) found no difference in IL-6 levels (taken at the time of the OGTT) between GDM and NGT women. Similar results were found by Simjak *et al.* (137) in 24 European pregnant women (GDM n=12, OGTT, IADPSG criteria) with normal BMI who examined IL-6 levels in the second and third trimester and post-partum and found no difference between GDM and NGT women.

Driven by the discordance in results, a recent systematic review by Amirian *et al.* (138) has explored the relationship between Il-6 and GDM in studies published between 2009 and 2020 and included 24 articles. The study highlighted the diversity of ethnicities involved, the different measurement methods but also the numerous criteria used to diagnose GDM (14 different diagnostic criteria) in the studies selected making significant research synthesis difficult. The common denominator for all studies, however, was the small sample size with the largest cohort in a study by Abdel Gader *et al.* (139) who found no difference in IL-6 levels between GDM and NGT women. Out of 24 studies, 16 found a positive association between IL-6 levels and GDM, the authors concluding that IL-6 can be used as a GDM biomarker. However, such a statement requires more scientific evidence. The heterogeneity of the studies to date involved in assessing the relationship between IL-6 and GDM is too high to be able to make any meaningful comparison.

Conceptually, IL-6 could be linked to GDM pathogenesis either through a higher degree of inflammation in GDM pregnancies (140), driven by increased subcutaneous or visceral adipose tissue (141) or increased IL-6 secretion by the placenta in GDM pregnancies (142). While IL-6 might prove to be a good GDM biomarker in the future, there are too many unanswered questions at present for such a claim. Larger studies with increased homogeneity in GDM diagnostic methods and criteria are required with serial IL-6 measurements in each trimester of pregnancy for identification of trimester

specific ranges, measurements of subcutaneous and visceral adipose tissue which might be the driver for its increase, and associations with adverse pregnancy outcomes.

g) Tumour necrosis factor (TNF)

TNF is an inflammatory cytokine family primarily secreted by monocytes/macrophages (143) with two main components TNF-α (also secreted from the placenta (144)) and TNF-β. The initial role for TNF was thought to be the death of tumour cells (145) but it was soon discovered that TNF plays an important role in inflammatory diseases (146), neurodegenerative disease (147), and depression (148). Given its pro-inflammatory effects, TNF has been identified as a marker of metabolic syndrome (149, 150), obesity (151) and insulin resistance (152, 153). Evidence suggests that TNF stimulates the secretion of IL-6 (154, 155), inhibits the secretion of adiponectin (156), induces apoptosis in adipose cells (157, 158) and inhibits the insulin receptor thus promoting insulin resistance (153, 159). A recent study by Alzamil *et al.* (160) examined the correlation between TNF-α and insulin resistance, T2DM and obesity in 128 Asian subjects (T2DM n=65). These authors found significantly higher TNF-α in T2DM subjects compared to controls, in obese subjects (T2DM or non-T2DM) compared to non-obese subjects and TNF-α levels were positively correlated with HbA1c levels and HOMA-IR highlighting the role TNF-α plays in the pathogenesis of insulin resistance and T2DM and the link with both obesity and glucose intolerance.

Guillemette *et al.* (161) studied TNF-α levels in both the first trimester of pregnancy and at the time of GDM diagnosis and its relationship to GDM in 756 pregnant women (GDM n=61, GCT/OGTT, IADPSG criteria). They found a positive association between TNF-α levels and BMI, adiponectin, and insulin levels in the first trimester and HOMA-IR, BMI, triglycerides, and fasting insulin levels in the third trimester. The authors also showed that TNF-α levels are strongly positively linked to insulin resistance and that it behaves differently during the OGTT in insulin sensitive and insulin resistant women.

Kirwan *et al.* (162) described longitudinal changes in TNF-α levels and the association with maternal insulin resistance in 15 women (GDM n=5, euglycemic-hyperinsulinemic clamp, Carpenter and Coustan criteria). They found that TNF-α in normal pregnancy, had lower levels in early pregnancy, increasing in late pregnancy paralleling insulin sensitivity changes, with higher levels in GDM women compared to lean NGT women. They also found that TNF-α was positively correlated with insulin sensitivity independent of BMI or glycaemic status. Proposed mechanisms for this were either increased TNF-α secretion by the placenta in GDM women and direct inhibition of the insulin receptor. This hypothesis is also supported by Desoye *et al.* (142). Syngelaky *et al.* (163) studied the link between first trimester TNF-α and the development of GDM in 1000 women (GDM n=200, random glucose/OGTT, WHO criteria) and found higher TNF-α levels in women with GDM compared to controls. The authors calculated that TNF-α can predict GDM development with an AUC of 0.82 but adding TNF-α levels to a multi-variable prediction model did not improve any of the estimated variables. While this was a large study, the GDM diagnostic method might have omitted milder cases of GDM that could have been included in the NGT group. A recent study by Wang *et al.* (63) explored TNF-α levels at GDM diagnosis in 110 Chinese pregnant women (GDM n=60, OGTT, ADA 2017 criteria) and found significantly higher TNF-α levels in GDM women compared to controls.

No correlation between TNF-α levels (samples taken in both first trimester and at GDM diagnosis) and GDM development was found by Georgiou *et al.* (38) in 250 women (GDM n= 14, OGTT, ADIPS criteria).

A systematic review and meta-analysis by Xu *et al.* (46) on the association between GDM and TNF-α levels, comprised 10 studies and despite the increased heterogeneity of the studies and missing confounders from the analysis, the authors found overall significant high levels of TNF-α in GDM pregnancies compared to controls independent of BMI.

The discrepancy in study results most likely lies in the different sample sizes, ethnicities, diagnostic methods and criteria and concentration limits employed. Also, fasting, exercise and stress influence TNF-α levels (164-168) and this needs to be taken into account when considering new diagnostic tests. While there is no doubt that TNF-α plays a role in the pathogenesis of GDM and insulin resistance, the actual predictability value of this biomarker is yet to be established.

2. Glycoproteins

a) Afamin

Afamin is a glycoprotein present in plasma, cerebrospinal fluid, ovarian and seminal fluid (169, 170), primarily expressed in the liver but also expressed in the brain and kidneys and its main role to bind and transport Vitamin E (169, 171) to peripheral tissues and organs. Studies looking at afamin levels in polycystic ovary syndrome (PCOS) cohorts, despite relatively small sample size, have found an association between afamin and insulin resistance and metabolic syndrome (172, 173). In a large multicentre study (n=20 136), Kollerits *et al.* (174) found that afamin was a strong predictor for the development of T2DM and strongly correlated with insulin levels, HOMA-IR and insulin resistance suggesting that afamin has the potential to be a biomarker for early prediction for future development of T2DM.

In pregnancy, afamin levels raise progressively with each trimester of pregnancy decreasing back to baseline post-partum with even higher levels in pregnancies complicated by pre-eclampsia or hypertension (175). Based on the previous findings which linked afamin with the development of insulin resistance and diabetes, it has been hypothesized that afamin might serve as a predictor for GDM. In two studies, Tramontana *et al.* (176, 177) explored the relationship between first trimester afamin and pregnancy complications in 4948 pregnant women and found significantly higher levels of afamin in women who subsequently developed GDM (n=207, IADPSG criteria) compared to NGT women. Afamin (cut-off value > 65 mg/l) was shown to be an independent predictor for developing GDM with a risk ratio of 2.07 (95% CI 1.33-3.22) and AUC of 0.66. Koninger *et al.* (178) looked at pre-pregnancy afamin levels in predicting GDM in a PCOS population (n=63, GDM n=29) and found higher afamin levels and HOMA-IR in women who developed GDM compared to controls with a strong positive correlation between afamin and HOMA-IR. The team showed that an afamin level of 88.6 mg/l identified GDM patients with a sensitivity of 79.3%, specificity of 79.4% and an AUC of 0.78 (95%CI 0.65-0.90). Ravnsborg *et al.* (179) studied potential GDM biomarkers in 270 first trimester samples with shotgun proteomics (GDM n= 135), diagnosed according to the Danish guidelines (180) and found higher afamin levels in GDM women compared with controls and that afamin could predict GDM diagnosis with an AUC of 0.67 (95% CI 0.53-0.81).

Koninger *et al.* (181) studied the predictive power of afamin in diagnosing GDM in both first trimester (n=110, of which 59 developed GDM) and second trimester of pregnancy (n=105, of which 29

developed GDM). GDM was diagnosed according to German Diabetes Association (DDG) and the German Association for Gynaecology and Obstetrics (DGGG)(182). They found that both first and second trimester afamin levels were higher in GDM women compared to NGT. Because this study comprised two different cohorts for first and second trimester samples, the samples were not taken longitudinally, therefore the team was not able to determine trimester-specific cut-off values for afamin levels. Another limitation of the study is the heterogeneity of the GDM diagnosis method and criteria used as not all women were screened with 75g 2-h OGTT and milder cases of GDM might have been missed.

Afamin is a very novel biomarker for GDM. It is not fully clear what is the exact mechanism through which afamin is linked to insulin resistance, metabolic syndrome, and glucose intolerance. In previous studies (170, 183) there has been observed no variation in afamin levels between fasting and non-fasting state, no circadian variation, no variation with menstrual cycle or gender variation suggesting that afamin is a stable biomarker for longitudinal measurements. There is not enough evidence to clearly state the true potential of afamin in predicting GDM but results to date are promising.

b) CD59

CD59 is an 18-20 kDa glycoprotein which is also known as membrane attack complex (MAC) inhibitory protein (MAC-IP) (184, 185). Its main role is to restrict MAC formation in the cell membrane thus preventing cell lysis and cell death. While CD59 is a protein bound to the cell membrane, soluble forms are present in the blood, urine, and saliva (186-188).

The link between diabetes complication and increased MAC deposits has been well documented (189-194). The first paper linking the increased MAC deposits in diabetes with CD59 inactivation was by Acosta *et al.* (195). They showed that in vitro CD59 exposure to glucose reduced its protection role leading to cell lysis. Building on this work, Qin *et al.* (194) measured CD59 levels in the red blood cells (RBC) of subjects with and without T2DM and found that there are significantly lower levels of CD59 in diabetic RBC compared to subjects without T2DM.

In 2013, Ghosh *et al.* (196) hypothesized that glycated CD59 (gCD59) levels might mirror glucose control in human subjects and developed a sandwich ELISA assay to identify plasma gCD59 which they tested initially in 24 participants with and without T2DM (T2DM n=14 HbA1c >48 mmol/mol) and then validated it in 190 subjects (T2DM n=100). gCD59 levels were significantly higher in the 14 individuals with T2DM from the initial testing set compared to controls and was strongly associated with HbA1c levels. gCD59 was able to identify T2DM with a sensitivity of 93%, specificity of 100% and AUC of 0.98. In the follow-up testing set, gCD59 levels were indeed higher in the T2DM group and positively associated with HbA1c levels with the test generating an AUC of 0.88. Continuing this work, Ghosh *et al.* (197) explored the link between gCD59 and glycaemic variables such as HbA1c (in 400 subjects (T2DM n=226) and glucose levels during the OGTT (n=109). The results supported previous findings, with gCD59 levels higher in diabetic vs. participants without diabetes and independently associated with HbA1c and with the 2-h glucose level on the OGTT. More so, the team also showed an acute response of gCD59 levels to insulin therapy in 21 poorly controlled subjects, with changes in levels in 2 weeks of treatment while HbA1c and fructosamine took 6-8 weeks to respond. This rapid turnover of values would have a particular importance in pregnancy and GDM where time is limited and in utero exposure to hyperglycaemia not without consequences.

Ma *et al.* (198) studied gCD59's capacity to predict GDM earlier in pregnancy (sample collected and OGTT performed <20 week's gestation) and the association with adverse pregnancy outcomes using

770 frozen samples collected as part of the Vitamin D and Lifestyle Intervention (DALI) study (Simmons D 2017). All the participants in the DALI study had a BMI ≥29 kg/ m^2 and underwent 3 OGTT s (<20 weeks GDM n= 207, 24-28 weeks GDM n= 77 and 35 weeks of gestation) and diagnosed according to the IADPSG criteria. gCD59 levels were higher in GDM women diagnosed <20 weeks of gestation independent of age, BMI or ethnicity and predicted the OGTT results < 20 weeks with an adjusted AUC of 0.86 (95% CI, 0.83-0.90). Restricting the analysis to the OGTT performed between 14-20 weeks of gestation the AUC was calculated at 0.90 (95% CI 0.86-0.93). Early gCD59 predicted GDM at 24-28 weeks with an AUC of 0.68 (95% CI 0.64-0.73). The team also found that higher gCD59 levels were associated with the risk of delivering an LGA baby. Some limitations of the study include the retrospective nature of the study, the inclusion of only high-risk women with a BMI ≥29 kg/ m^2 and low ethnic diversity.

In 2017, Ghosh *et al.* (199) explored the association between gCD59 and the results of the GCT, the results of the OGTT and the prevalence of large for gestational age (LGA) babies in 1000 pregnant women at 26 weeks of gestation (500 women passed the GCT and were controls and 500 women failed the GCT and underwent a 3-h OGTT). gCD59 was 8.5 times higher in women who failed the GCT compared to those who passed it and 10 times higher in women who were diagnosed with GDM (n=127) on the 3-h OGTT (Carpenter and Coustan criteria). gCD59 predicted GCT failure with a sensitivity of 90%, specificity of 88% and adjusted AUC of 0.92 (95% CI 0.88-0.93) and predicted the development of GDM compared to controls with a sensitivity of 85%, specificity of 92% and adjusted AUC of 0.92 (95% CI 0.77-0.91), independent of age, BMI, ethnicity of history of diabetes. More so, the team also identified significantly higher gCD59 levels in women who gave birth to an LGA baby. There are some limitations to this study including its observational nature, the use of GCT (which might not be done in the morning), the 3h-OGTT and the Carpenter and Coustan criteria for GDM diagnosis arguably missing milder cases of GDM.

gCD59 is a very promising biomarker that has shown a lot of potential in the diagnosis and early diagnosis of GDM and prediction of LGA born infants. The rapid turnover of values and the lack of need of fasting certainly is a significant advantage for a pregnancy biomarker. However, there are still unanswered questions such as: what are the trimester specific cut-off values? Are there any discrepancies in cut-off values amongst different ethnicities? Could early pregnancy gCD59 predict the 24-28 OGTT results in a BMI diverse population? Larger prospective studies are required to answer these questions and one such study is currently underway (200).

c) Human chorionic gonadotropin (hCG)

HCG is a glycoprotein hormone, mainly secreted by the placenta, whose main role is in embryo implantation and control of embryogenesis (201). Recently, however, Ma *et al.* (202) have shown that hCG influences insulin sensitivity and induces adipocyte mediated inflammation and consequently might contribute to GDM pathogenesis. The beta isoform of hCG (β-hCG) is part of the first trimester screening for foetal aneuploidy.

Sirikunalai *et al.* (203) retrospectively studied the link between β-hCG levels and adverse pregnancy outcomes including GDM in 13,620 pregnant Thai women and found that high first trimester β-hCG levels were associated with a decreased risk of developing GDM. This finding was not sustained in the second trimester. While this study had a large sample size, a high number of women had incomplete data and due to the retrospective nature of the study, adequate adjustments and multivariate analysis

could not be done due to the lack of absent confounders. Ong *et al.* (204) measured β-hCG levels between 10-14 weeks of gestation in 5584 pregnant women. Women were diagnosed with GDM with a 2-h OGTT and diagnosed according to the 1980 WHO criteria (205). The team found significantly lower β-hCG levels in women that developed GDM (n=49) compared to NGT suggesting that first trimester β-hCG could predict second trimester GDM diagnosis. A limitation of this study and an explanation for the small number of GDM cases detected is the GDM diagnosis criteria used which would only identify severe cases of GDM, with milder cases not being included in the study. The use of IADPSG criteria in this cohort would have led to a more representative sample of the general population and ease the generalizability of results. Xiong *et al.* (206) retrospectively analysed β-hCG levels in 1596 cases, 11 days after single blastocyst transfers (assisted reproduction) with 370 live births and found significantly higher rates of GDM (GDM total n=61) in women with low levels of β-hCG compared to women with high levels of β-hCG. Beyond the retrospective nature of the study, no information is provided on the GDM diagnosis and criteria used; the number of GDM women in the low level β-hCG subgroup is quite small (n=5) and insufficient for a robust comparison. Controversially, Yue *et al.* (207) measured β-hCG levels between 14 and 20 weeks of gestation in 8333 pregnant Asian women, 1336 of which developed GDM (ADA criteria) and found high β-hCG levels are an independent risk factor for the development of GDM. A possible explanation for this discrepancy might be the more advanced week of gestation when the sample was collected with reactive β-hCG levels secreted by a hypoxic placenta as a response to hyperglycaemia; also, the overall BMI of the cohort was very low compared to previous studies.

In a retrospective study, Tul *et al.* (208) measured first trimester β-hCG levels in 1136 Caucasian women (GDM n=27) and found lower yet not statistically significant levels in women who developed GDM. In this cohort, GDM was diagnosed with the 3-h OGTT and given the number diagnosed, would equate to a GDM prevalence of 2.37% which is extremely low compared with the overall European GDM prevalence. It is unclear from the paper whether this low prevalence is due to missing data. However, these results are supported by Savvidou *et al.* (209) who retrospectively assessed β-hCG levels at 11-13 weeks of gestation in 42,102 pregnant women. GDM (n=779) was diagnosed with a 2-step approach, the women undergoing an OGTT only if the random plasma glucose at 24-28 weeks of gestation was higher than 6.7 mmol/l. The team found no difference in β-hCG levels between women who developed GDM and NGT. No correlation in first trimester β-hCG levels and GDM development was also found in Beneventi *et al.* (210) (GDM n=228, GCT/ 100g 3-h OGTT, Carpenter and Coustan criteria) or Sweeting *et al.* (211) (GDM n=248, OGTT, Australian Diabetes in Pregnancy Society criteria (116)).

There is quite a high degree of heterogeneity in design, populations and GDM diagnosis methods leading to inconsistent results. None of the studies looked at the longitudinal trend of β-hCG levels in the first and second trimester and GDM diagnosis which would have clarified the cause of variable levels – low levels in the first trimester secondary to compromised placentation or reduced placental mass; high levels in the second trimester of pregnancy secondary to hyperglycaemia induced placental hypoperfusion. It seems, however, that studies which involved a higher-risk population for the development of GDM (assisted reproduction, GDM diagnosis criteria that identifies more severe cases of GDM etc.) were more likely to find an association between β-hCG levels and GDM. Perhaps single β-hCG levels could be used to identify a possible at risk for GDM population that should be adequately followed up and screened. However, current evidence does not support this, and future more consistent studies are required.

d) Sex Hormone Binding Protein (SHBG)

SHBG is a glycoprotein produced by the liver, brain, uterus, testes and placenta (212) and its main role is to bind and transport biologically active androgens and oestrogens (213). SHBG is linked to adipose tissue with lower levels in obese subjects (214) which increase when weight loss is achieved (215, 216). SHBG has also been linked to insulin resistance (217, 218), metabolic syndrome (219-221) and the development non-alcoholic fatty liver disease (NAFLD) independent of BMI and T2DM (222-224). Potential mechanisms suggested for this are either a direct effect of insulin on SHBG production (225, 226) or fat accumulation in the liver and/or increased hepatic triglycerides levels leading to decreased SHBG gene expression (227-229). The role of SHBG in GDM diagnosis has been explored in numerous studies at different time points during pregnancy, including pre-pregnancy with overall promising results.

Veltman-Verhulst *et al.* (230), in a prospective study, measured SHBG in 50 women with PCOS prior to pregnancy (median 35 weeks) following fertility treatment. GDM diagnosis was made based on a 3-h OGTT at 24-28 weeks of gestation (GDM n=21). SHBG levels were significantly lower in the GDM group compared to NGT and, with a cut-off level of 58.5 nmol/L, SHBG could predict GDM with a sensitivity of 81.0%, specificity of 82.8% and AUC of 0.86 (95% CI 0.75-0.97). Hedderson *et al.* (231) studied the link between pre-pregnancy (median 6.2 years) levels of SHBG and the subsequent development of GDM in a case-control study (GDM n=267, 3-h OGTT, Carpenter and Coustan criteria) and found a significantly lower level of SHBG in women who developed GDM independent of GDM risk-factors. This study showed that SHBG levels measured years (min. 6 years) prior to pregnancy can predict the development of GDM even in very low risk women and this is of high clinical importance. Study limitations include the lack of longitudinal anthropomorphic data, additional SHBG measurements during pregnancy and the lack of markers of visceral adiposity. Badon *et al.* (232) measured SHBG in a case control study within a cohort of 4098 pregnant women (GDM n=267) at a median 7 years prior to pregnancy and similar to previous studies found significantly lower SHBG levels in women who subsequently developed GDM compared to controls with a predictive value of 0.71.

In a longitudinal study, Li *et al.* (233) measured SHBG levels in 321 women (GDM n=107) in all 3 trimesters of pregnancy. SHBG levels increased progressively with the trimester of pregnancy in both GDM and NGT groups with significantly lower levels in GDM compared to controls in the first trimester. This significance disappeared in late pregnancy suggesting that perhaps the best time to measure SHBG is early in pregnancy as lifestyle changes or treatment for GDM in late pregnancy might influence SHBG levels. They also found SHBG levels to be negatively associated with markers of insulin resistance. This study confirmed the results of a previous study by Smirnakis *et al.* (234) who measured SHBG levels in 145 women (GDM n=37, GCT, ACOG criteria (235)) at 11 and 17 weeks of gestation and found lower levels in women who subsequently developed GDM compared to controls, with stronger association at 11 weeks of gestation. Caglar *et al.* (236) found that a SHBG cut-off level of 97.47 nmol/L (at 13-16 weeks of gestation) could predict GDM with a sensitivity of 46.7%, specificity 84.1% and AUC 0.67 (95% CI 0.55-0.79) while Maged *et al.* (237) using a first trimester SHBG cut-off value of 211.5 nmol/L calculated a sensitivity of 85.2%, specificity 37% and AUC of 0.69.

Tawfeek *et al.* (238) in a case control study, measured SHBG levels at the time of the OGTT at 24-28 weeks of gestation (GDM n=45, 75g 2-h OGTT, IADPSG criteria) and found significant lower SHBG levels in the GDM group compared to NGT. At a cut-off value of 50 nmol/L SHBG could identify GDM with a sensitivity of 96%, specificity of 95% and AUC of 0.91 (95% CI 0.82-1.) A similar study in the same population by Siddiqui *et al.* (239) (GDM n=53, OGTT, ADA criteria) also found lower levels of SHBG in GDM women compared to controls but only in nulliparous women with a positive correlation with

gestational age. Limitations of both studies include the small sample size with a relative high BMI and ethnically confined to Asian participants which tend to have a higher prevalence of metabolic syndrome (240).

McElduff *et al.* (241), however, found no difference in SHBG levels between GDM and NGT groups in their cross-sectional study which included 220 pregnant women (GDM n=642, GCT/OGTT, Carpenter and Coustan criteria). Despite a robust study methodology, similar to previous studies, the main reason for these discordant results lies in the difference in population characteristics and diagnostic method.

SHBG is a straightforward, low-cost test that does not require fasting (242) and has no diurnal variation (243). This test has shown some promise as a predictor of GDM when used prior to or in the first trimester of pregnancy and this might be because the difference in insulin resistance markers reduces as the pregnancy progresses. Catalano *et al.* (244) found higher levels of insulin resistance in the first trimester of pregnancy in women with NGT in the first trimester who eventually developed GDM compared to women with NGT all throughout. In most studies, the link between SHBG and GDM was independent of subcutaneous adiposity suggesting, perhaps, that liver adiposity would be a better marker for SHBG production (245). But further studies are required to measure SHBG levels, markers of insulin resistance, of glycaemic control and hepatic steatosis for a better understanding of the role of SHBG in GDM. Beyond that, however, we cannot ignore the positive results in studies assessing pre-pregnancy and first trimester predictability of GDM development which would allow for early interventions on the modifiable factors involved in GDM development. Standardization of pre-pregnancy and trimester specific cut-offs, GDM diagnosis cut-off value, exploration of the variability of levels in different populations and standardisations of assays, would make SHBG a very promising marker for the early diagnosis of GDM.

4. Other Proteins

a) C-reactive protein (CRP)

CRP is an acute phase protein secreted and released by numerous cells in the context of inflammation (246, 247). Obesity, which is a pro-inflammatory state, is a known risk factor of GDM. CRP is a non-specific marker which may be elevated in settings such as infection or obesity in the absence of GDM (248-250). High levels of CRP have been described in association with insulin resistance and metabolic syndrome (251-254). Numerous studies have found an association between obesity and high CRP levels independent of insulin resistance (255-257). Therefore, it is biologically plausible that inflammatory markers such as C-reactive protein (CRP) could be a promising biomarker of GDM.

Alamolhoda *et al.* (258) prospectively studied the relationship between first trimester CRP levels and the risk of developing GDM in 120 pregnant women (GDM n=11, OGTT) and found a significant difference between GDM women and controls independent of BMI. The sample size however was small and the cut-off value for fasting glucose at diagnosis was 7mmol/l (126 mg/dl) which selected only the more severe cases, a fasting glucose of 7mmol/l being the cut-off diagnostic value for T2DM. In a case control study involving 372 women (GDM n=124, OGTT, WHO criteria), Savvidou *et al.* (259) also found higher first trimester CRP levels in women who subsequently developed GDM compared to controls. While the sample size was larger, similar to the previous study the fasting glucose cut-off

was 7mmol/l (126mg/dl). Kansu-Celik *et al.* (83) investigated first trimester high-sensitivity CRP (hsCRP) as a biomarker for GDM diagnosis in 88 pregnant women (GDM n=29, GCT/OGTT, Carpenter and Coustan criteria) and found that hsCRP was significantly higher in women who subsequently developed GDM independent of BMI with a sensitivity of 86.2%, specificity of 50.8% and AUC of 0.70 (95% CI 0.59-0.81) at a cut-off value for hsCRP of 4.65 ng/ml. Wolf *at al.* (260) measured first trimester CRP levels in 131 women (GDM n=43 , GCT/OGTT, ADA criteria) and found that women diagnosed with GDM had higher CRP levels in the first trimester of pregnancy with a strong positive correlation between CRP levels and 1-h post glucose load levels and systolic blood pressure. The team also found that the addition of BMI in a multivariate model attenuated the correlation between CRP and GDM diagnosis suggesting the influence of adipose tissue on CRP levels. Some of the limitations of this study include the small sample size, the single time point measurement of CRP and the lack of additional adiposity markers beyond BMI (such as waist circumference, visceral fat).

Alyas *et al.* (261) measured high-sensitivity CRP (hsCRP) levels at 14-18 weeks of gestation and 24-28 weeks of gestation in 158 women (GDM n=58, OGTT, IADPSG criteria) and found significantly higher CRP levels at both time points in women diagnosed with GDM compared to controls. No analysis on BMI category was done in this study.

Conflicting results are found in a cross-sectional study by Korkmazer *et al.* (262) who measured hsCRP at GDM screening time point in 116 women who underwent a GCT followed by an OGTT and were classified in GDM (n=39, failed GCT and OGTT, Carpenter and Coustan criteria) and glucose intolerant (n=37, abnormal GCT, normal OGTT) and controls. The team found no differences in hsCRP levels between the 3 groups. The sample size was small which might explain these findings and no analysis was done between the GDM and glucose intolerance groups together and the controls. Corcoran *et al.* (263) evaluated hsCRP in 225 pregnant women with one or more risk factors for GDM in the first trimester (46 of which developed GDM, OGTT, IADPSG criteria) and found no difference in hsCRP levels between the GDM and the control group findings also supported by Adam *et al* (264) .Retnakaran *et al.* (249) measured CRP levels at the time of the OGTT in 180 women (GDM n =39, impaired glucose tolerance n=48, GCT/OGTT, National Diabetes Data Group (NDDG) criteria (265)) and found no association between CRP levels and pregnancy glycaemic status but did describe a strong association between CRP levels and pre-pregnancy BMI and fasting glucose.

A systematic review by Amirian *et al.* (266) investigated the association between CRP and GDM diagnosis and included 31 articles. Even though no meta-analysis was done in this study due to the lack of clinical data, the authors found a positive association between high CRP levels and GDM development in 20 studies (CRP n = 8 articles, hsCRP n=12 articles) while 11 studies (CRP n= 6 articles, hsCRP n=5 articles) did not identify any correlation. The main reasons for these discrepancies are the variations in diagnostic methods and criteria, difference in sample size and population characteristics, different methods to quantify CRP levels or the lack of adjustment for BMI and other confounders.

While CRP/hsCRP shows some potential, the literature shows inconsistent and contradictory data with most studies having small sample size cohorts. This arises from the wide arrays of methodology and study population features. A big disadvantage of using CRP as a diagnostic biomarker is its non-specificity as a high result will possibly lead to a wide range of investigations, some unnecessary, increasing costs and the pregnant woman’s stress levels. Further research is required to clarify the correlation between adiposity (subcutaneous or visceral) and CRP levels and the reflection of this association in the glycaemic status. CRP might play a more meaningful role as a risk assessment tool for GDM screening rather than GDM diagnosis.

b) Nesfatin-1

Nesfatin-1, initially described in 2006, is a neuropeptide produced primarily by the hypothalamus and brain stem and its main role is in food and water intake regulation, control of appetite with anorexigenic properties (267). Research on animal models (268, 269) found that intravenous nesfatin-1 regulated fatty acid metabolism, reduced insulin levels, improved insulin sensitivity and reduced blood glucose levels in mice. Li *et al.* (270) measured nesfatin-1 levels in healthy adults, and adults with T1DM and T2DM and found significantly lower nesfatin-1 levels in individuals with T2DM compared to controls (not valid for T1DM) independent of BMI and no significant change in levels during the OGTT. A systematic review and meta-analysis by Zhai *et al.* (271) studied the association between T2DM and nesfatin-1 and comprised 7 studies and 627 participants (T2DM n=328) with 6 out of 7 studies being carried out in China. The authors found significantly higher nesfatin-1 levels in newly diagnosed T2DM compared to controls; however, overall, when all participants were included, there was no significant association between T2DM and nesfatin-1. All the studies included had small size numbers of participants, and there was no sub-analysis on duration of diabetes, BMI sub-category, insulin resistance markers or glycaemic control (HbA1c) on or off treatment.

Given the anorexigenic, the anti-hyperglycaemic effect of nesfatin-1 and moderate evidence of association with T2DM, studies have also assessed a possible implication of nesfatin-1 in GDM. A prospective study by Kucukler *et al.* (272) measured nesfatin-1 levels at 24-28 weeks of gestation in 79 pregnant women (GDM n= 38, GCT/OGTT, ADA criteria) and found significantly lower nasfatin-1 levels in the GDM group compared to NGT at diagnosis. Nesfatin-1 was negatively associated with BMI, fasting glucose, and HOMA-IR. Measurements at 24-28 weeks of gestation were also taken by Ademoglu *et al.* (273) in 70 pregnant women (GDM n=30, GCT/OGTT, Carpenter and Coustan criteria) and similar to previous studies found lower nesfatin-1 levels in women with GDM compared to NGT women independent of age, BMI, fasting glucose and HOMA-IR at diagnosis. No correlation was found between nesfatin-1 and fasting glucose, BMI, or markers of insulin resistance. In a larger study, Mierzynski *et al.* (274) measured nesfatin-1 levels at 24-28 weeks of gestation in 237 women (GDM n=153, OGTT, WHO criteria) and found lower nesfatin-1 levels in GDM subjects compared to controls. There was a positive association between nesfatin-1 levels and BMI, glucose levels and gestational age.

Nesfatin-1 is a recent biomarker with only a few, small size studies exploring its role in GDM pathogenesis. It is a possibility that nesfatin-1 could act as an anti-diabetic agent by enhancing insulin action/ secretion, reducing glucose levels, and reducing food intake and a decrease in nesfatin-1 levels in pregnant women might lead to insulin resistance and GDM. Another theory is that insulin resistance and hyperinsulinemia might inhibit the secretion of nesfatin-1. There are no studies assessing nesfatin-1 levels dynamically in normal pregnancy or any studies measuring first trimester levels in women who will develop GDM. Nesfatin-1 has been shown to be an important component of glucose dysregulation pathway in both GDM and T2DM, but the exact mechanisms and the exact cut-off values required for accurate interpretation requires substantial future studies.

c) Pregnancy-associated plasma protein A (PAPP-A)

PAPP-A is a zinc-binding matrix metalloproteinase secreted by the trophoblast and can be measured as early as 28 days of pregnancy (275). PAPP-a has been used as a screening test in the first trimester of pregnancy for aneuploidy and for identifying certain adverse pregnancy outcomes (276, 277). Through its properties, PAPP-A increases insulin-like growth factor 1 (IGF-1) bioavailability through its

cleavage from the IGF binding protein -4 suggesting a possible link between PAPP-A and insulin sensitivity. Pellitero *et al.* (278) found lower PAPP-A levels in diabetic patients compared to controls with a negative association between PAPP-A levels and HbA1c. Also, it has been documented that TNF-α (inflammatory cytokine with a role in insulin resistance) strongly stimulates PAPP-A secretion (279). Therefore, it has been hypothesized that low PAPP-A levels are linked to insulin resistance in pregnancy through low levels of IGF-1 that led to hyperinsulinemia.

Lovati *et al.* (280), in a case-control study, explored the association of first trimester levels of PAPP-A and GDM development in 673 Caucasian pregnant women (GDM n=307, 100g 3h OGTT/ 75g 2g OGTT) and found significantly lower levels in women who were diagnosed with GDM and even lower levels in women that required insulin therapy. PAPP-A (in addition to clinical risk factors) could predict GDM development with a sensitivity of 81.4%, specificity of 50.5% and an AUC of 0.70 (95% CI 0.66-0.73). Similar results are found by Ramezani *et al.* (281) who prospectively measured first trimester PAPP-A in 286 Middle Eastern women (GDM n=45, OGTT, IADPSG criteria) and found significantly lower PAPP-A levels in women who developed GDM. PAPP-A could identify the future development of GDM with a sensitivity of 73.3%, specificity of 57.3% and AUC of 0.61. This study however did not record certain variables and adequate adjustments in the analysis have not been made. A similar design study (282) in a comparative size Asian cohort (GDM n=45, OGTT, IADPSG criteria) found that PAPP-A could predict GDM with a sensitivity of 72.5%, specificity of 82.3% and AUC of 0.86.

In a large retrospective study, Snyder *et al.* (283) studied clinical and biomarker models for early GDM diagnosis in 66,687 (GDM n=4,874) in ethnically and racially diverse pregnant women. Samples were collected in both first and second trimesters of pregnancy. The team found significant lower levels of PAPP-A in women who subsequently developed GDM compared to controls with no difference between groups in second trimester samples. The addition of PAPP-A to the clinical risk prediction model only slightly improved the prediction accuracy of the model. These findings are supported by other studies (284, 285) who did not find a significant change to AUC by the addition of PAPP-A to the clinical prediction model. While this was a large study, it only included nulliparous women, there was no information on the GDM diagnostic method or criteria used and there was no BMI category sub analysis. These findings suggest that PAPP-A could be useful as a first trimester of pregnancy predictor for GDM development with limited utility as in GDM diagnosis in the second trimester of pregnancy.

A systematic review and meta-analysis by Donovan *et al.* (286) included 13 studies and 83,921 subjects (GDM n=3,786). While the study identified a high degree of heterogeneity mostly due to the analysis method, the GDM diagnostic criteria and the ethnicity of subjects involved, the overall analysis and sub analysis identified significantly lower first trimester PAPP-A levels in women who developed GDM compared with controls with even lower levels in GDM women diagnosed prior to 24 weeks of gestation. This correlation was not as strong in women of Asian origin. The correlation between PAPP-A levels and the degree of glucose intolerance in GDM was also highlighted by Wells *et al.* (287) who found lower levels of PAPP-A in women with early GDM diagnosis compared to late diagnosis and the lowest levels of PAPP-A in women diagnosed with T2DM.

Research to date has not clarified if low levels of PAPP-A promote or are rather the result of impaired glucose metabolism and insulin resistance. The reduced observed levels of PAPP-A in GDM pregnancies might reflect a defect in placentation or placental insufficiency encountered in GDM pathology. The studies, however, have consistent results with overall lower PAPP-A first trimester levels in GDM pregnancies. And these findings are also supported by studies exploring exosomes profiles as a biomarker for GDM diagnosis (288). Despite the variable reported predictive value across the literature which is mostly due to patients' characteristics, sample size and GDM diagnostic criteria variability, PAPP-A is routinely assessed in first trimester abnormalities screening and it may identify

women at high risk for early development of GDM. Further prospective studies are required to elucidate the clinical utility of this biomarker on its own or incorporated in risk identification models.

d) Retinol binding protein 4 (RBP4)

RBP4 is secreted mainly by the liver and adipose tissue. Its main role is to transport retinol (Vitamin A) from the liver to the peripheral tissues (289). RBP4 also has a role in inflammation and adipose tissue dysfunction (290), in increasing hepatic glucose output, in reducing insulin signalling in the muscle and in increasing insulin resistance (291).

Jin *et al.* (292) measured RBP4 levels in the first and second trimester of pregnancy in 270 women (GDM n=135, IADPSG criteria) and found that GDM women had higher first trimester levels of RBP4 compared to NGT women, that higher levels of RBP4 were associated with a higher risk of developing GDM and that RBP4 levels in both trimesters were positively independently associated with markers of insulin resistance. Yuan *et al.* (293) measured a panel of biomarkers with potential to diagnose GDM in 359 pregnant women (GDM n=86, IADPSG criteria) at 16-18 weeks of gestation. RBP4 was significantly higher in women that developed GDM compared to NGT. The study also found that RPB4 (cut-off value >30.45 µg/mL) could predict the development of GDM with a sensitivity of 63.6%, specificity of 75% and AUC 0.72 (95% CI 0.64-0.79) and that RPB4/adiponectin ratio (cut-off >0.37) could predict GDM with a sensitivity of 81.8%, specificity of 75.6% and AUC of 0.80 (95% CI 0.73-0.87). A retrospective study by Du *et al.* (294) found that second trimester RBP4 levels were significantly higher in the GDM group (n=194, OGTT, IADPSG criteria) compared to NGT women with a strong association between RBP4 levels, insulin levels and HOMA-IR. The authors found that RBP4 (cut-off levels 34.84 µg/mL) can predict GDM with a sensitivity of 79.4%, specificity of 79.1% and AUC of 0.87 (85% CI 0.83-0.92).

Discordant results are found in a study by Khovidhunkit *et al.* (295) who measured RBP4 in 532 women (GDM n= 171, GCT/OGTT, Carpenter and Coustan criteria) between 24-28 weeks of gestation and found no difference in RBP4 levels between GDM and NGT women and no correlation with insulin levels and HOMA-IR. They did find a positive independent association with fasting triglycerides and weight gain in pregnancy. All the women in this study were of Thai ethnicity with a low/normal BMI which might account for the discordant result with previous studies along with different diagnosis method (OGTT/GCT) and different sampling gestational week.

Two meta-analyses (296, 297) studied the link between RBP4 and GDM. Huang *et al.* (296) included 14 studies (GDM n=884) and found that RBP4 levels were significantly higher in women with GDM compared to NGT women, independent of age or BMI. On a subgroup analysis, however, this significant difference was only maintained for Asian populations with no difference in levels in non-Asian populations. This study had a low probability of bias but a high degree of heterogeneity; the link between RBP4 levels and GDM varied with GDM diagnostic criteria (WHO criteria- higher levels of RBP4 in GDM patients compared to controls; ADA criteria – no difference in levels between groups) and varied with different assays used for RBP4 determination. These findings are supported by a meta-analysis by Jia *et al.* (298) who found higher levels of RBP4 in patients of Asian ethnicity compared to controls but not in patients of European ethnicity compared to controls. A meta-analysis by Hu *et al.* (297) pooled results from 14 studies (case-control) (GDM n= 647) and found that RBP4 levels taken between 24-28 weeks of gestation were associated with the risk of developing GDM. Similar to the previous meta-analysis, there was no difference between groups in studies that used the ADA criteria

for GDM diagnosis suggesting that higher glucose levels on the OGTT are associated with higher RBP4 levels.

There are contradictory findings in the literature with some studies finding a positive link between RBP4 and GDM (295, 299-302) (Asian population) while other studies could not determine an association (303-306) (non-Asian population) and there is scarce evidence on first trimester of pregnancy RBP4 and the risk of GDM. If ethnicity plays such an important role in RBP4 levels, this requires further evaluation in much larger studies with multi-ethnic participation.

It is unclear if the free or bound RBP4 (or total) serves as a better predictor for GDM. Some studies (304, 307) suggest that, in fact, RBP4/transthyretin (RBP s binding protein) ratio might be a better marker for insulin resistance and GDM compared to RBP4 alone.

A possible cause for discordant results is the different assays used in the measurement of RBP4. Graham *et al.* (308) measured RBP4 levels in subjects with insulin resistance and glucose intolerance and insulin sensitive subjects with NGT using 3 commercial assays and a quantitative wester blotting assay and found substantial inconsistency amongst the results with enzyme immunoassays underestimating RBP4 levels concluding that western blotting is the most reliable method for measuring RBP4.

Discussion

This scoping review highlighted the large number of biomarkers described in the literature investigated for their potential to identify GDM and described 15 protein biomarkers selected based on higher number of citations in very recent publications in our literature search.

The current screening methods for GDM pose several issues

1. Universal Vs Selective screening
   Numerous studies (309-312) have shown that universal screening offers a significant advantage over selective screening by identifying all GDM cases, enabling timely lifestyle interventions and treatment, leading to a reduction in GDM associated adverse events. Proponents of selective screening invoke reduced complications associated with milder cases of GDM that would be diagnosed through universal screening as the argument in addition to the increased healthcare costs of screening. It is well known that GDM poses a long-term threat to the health of the mother and child through chronic metabolic diseases in a young population that will considerably increase the lifetime overall healthcare costs. Identifying GDM and intervening to prevent long term issues has been shown to be cost effective. A systematic review by Mo *et al.* (313) included 10 economic evaluations on different GDM screening strategies and found that universal screening is more likely to be cost-effective compared to selective screening. This finding is supported by other studies (314, 315).
   Beyond the costs, the focus should be on the accurate identification of cases and prevention of adverse outcomes. In a Malaysian population, Idris *et al.* (309) found that when universal screening was employed, the OGTT yielded a sensitivity of 83.5% and specificity of 82.6%. When selective screening was employed the sensitivity and specificity of the OGTT were lower 76.1% and 60.9%, respectively leading to 23.8% of women with GDM to be missed. In a European cohort, Miaihle *et al*. (316) showed that selective screening would have missed one-sixth of GDM cases. Similar results were found by Cosson *et al.* (317) in a retrospective study

which included 18,775 pregnancies. The authors found that applying selective criteria for screening would lead to 34.7% of GDM cases being missed.

It was suggested that low-risk women with GDM would have a good prognosis and not being diagnosed with GDM would not lead to adverse pregnancy outcomes. The literature has conflicting data with some studies finding no benefit on adverse pregnancy outcomes when universal screening was applied (316, 318) while others have shown significant benefits (319-321).

As selective screening would miss a significant number of women with GDM, and as universal screening has been shown to be cost-effective compared to selective screening most international bodies now recommend universal screening. One of the barriers to the implementation of universal screening are the logistics of performing OGTT in the entire pregnant population. Accurate biomarkers as an alternative to the OGTT would allow universal screening to become a reality.

2. Time of screening

Standard GDM screening occurs between 24-28 weeks of gestation. This arguably leaves a very narrow window for intervention. Some studies suggest that women that develop GDM early in pregnancy (<12 weeks of gestation) have outcomes comparable to women with pre-pregnancy diabetes despite treatment (322, 323) while others found that early diagnosis and treatment may lead to a reduction in LGA (324). Research has focused on the differences in pregnancy outcomes between women with GDM diagnosed in the first and late second trimester, but we also need to consider the possible long-term impact of the foetal intrauterine exposure to hyperglycaemia between onset and diagnosis and while this may not be obvious at birth, the hyperglycaemia-triggered foetal metabolic programming can lead to metabolic syndrome, insulin resistance and obesity in young adults (325). One of the concerns with early screening is that while some women with more severe forms of GDM will be diagnosed early in pregnancy, others will only develop glucose abnormalities later in pregnancy and will require another test at 24-28. A single non fasting biomarker would allow repetitive testing through pregnancy to facilitate rapid identification of hyperglycaemia.

3. Diagnostic criteria

As illustrated, it is difficult to make a meaningful comparison between studies when different criteria are employed to diagnose GDM. Studies that use a two-step approach with higher glucose cut off thresholds will select a population with more severe forms of GDM and the results cannot be extrapolated to the general population. Using a common new diagnostic biomarker will lead to harmonisation of GDM diagnosis and all data synthesis to be performed

4. OGTT

Women are currently diagnosed with GDM using an OGTT. This test is unreliable with poor reproducibility and high vulnerability to external and internal factors (15). More so, the OGTT does not identify the continuous correlation between hyperglycaemia in the mother and pregnancy complications, and possibly omits milder forms of glucose abnormalities that may identify pregnancy risks. Studies to date evaluating novel biomarkers as diagnostic tests/tools in GDM use the OGTT as the gold standard for comparison. But how valid are the results if the comparator test is flawed? It may be more accurate to assess the predictive power of the biomarker to identify adverse pregnancy outcomes Hyperglycaemia is not the only contributor to adverse pregnancy outcomes and other metabolic factors, such as adiposity, dyslipidaemia, inflammation, should be considered when considering a test (or panel of tests) with the highest potential to identify pregnant women at risk. Changing the focus from the glucose value to the outcomes we need to prevent and the factors contributing to them will bring the research community closer to identifying the next screening test. This would start

with a consensus on what are the outcomes we are aiming to prevent (macrosomia, LGA, hypertensive disorders of pregnancy, polyhydramnios etc), a reflection on the pathophysiology of the outcome and a reassessment of novel biomarkers not in their capacity to identify an out-of-range glucose value but in their capacity to capture the cumulus of mechanisms that lead to adverse pregnancy outcomes.

The COVID-19 pandemic has highlighted the difficulties in performing the OGTT in such a challenging environment, leading to OGTT screening being terminated and a high number of women being undiagnosed. This further emphasizes the urgent need for a single non-fasting sample biomarker test that could be performed in a family practice setting (GP) rather than a hospital setting.

Identifying a biomarker for the accurate diagnosis of GDM would have numerous practical benefits. A single blood test would reduce the appointment length, would enable a greater number of women to be screened (aiming for universal screening) and would enable the test to be performed in a non-hospital setting. A test that does not require fasting not only would considerably reduce the discomfort a pregnant woman experiences but would also enable appointments for sample collection throughout the day thus increasing the number of women being screened. A test that does not require glucose loading, reducing adverse experiences such as nausea, vomiting, pre-syncopal episodes, would considerably increase compliance with testing. Studies assessing the robustness of novel biomarkers to pre-analytical and analytical variables, time-to result analysis and cost-effectiveness analysis will be required in the future.

Biomarker research has grown exponentially in recent years out of a need for more accurate, more direct measurement of disease and has proven to be a powerful tool in the understanding of physiology and pathophysiology. But while biomarkers have many advantages, much like other tests, there are several things that need to be considered and assessed when conducting biomarker research: 1) interindividual variability; 2) intraindividual variability; 3) sample collection/transportation/storage; 4) biomarker validity; 5) predictive power; 6) confounding variables; 7) normal ranges and 8) cost (326).

The main limitation of this study lies in the nature of its design. Scoping reviews do not formally evaluate the quality of evidence and the evidence is collected from studies of different designs and methodology. Therefore, the data collected cannot be presented in a systematic way, but instead it gives an overall view of the existent literature. The scope of this review was to offer the reader an insight on the vast number of molecules studied in relation to GDM diagnosis and the potential diagnostic value of the selected novel protein biomarkers. This approach, however, led to a certain degree of selection bias. Another limitation of the study is the time lapse between the systematic review search and the publication of the manuscript leading to very recent research not being included in our study.

Perspectives

Given the limitation mentioned above, several protein biomarkers did not fulfil our inclusion criteria and were not included in the discussion. Therefore, we want to give a short overview of promising protein biomarkers evaluated for GDM prognosis/diagnosis in articles published between January 2020 and March 2021 that should be considered in the future.

Secreted Frizzled-Related Protein 4 (sFRP4) has been shown to play a role in glucose metabolism reflecting islet inflammation and impaired insulin secretion (327). Schuitemaker *et al.* (328) found

significantly higher first trimester levels of sFRP4 in women who subsequently developed GDM (n=50, diagnostic criteria fasting glucose ≥ 7.0 mmol/L, 2 h glucose ≥ 7.8 mmol/L) compared to controls and a predictive capacity expressed as AUC of 0.60 (95%CI 0.50-0.70). The correlation between sFRP4 and GDM is supported by other studies (329, 330)

Amini *et al.* (331) studied alfa-fetoprotein (AFP) as a predictor for GDM in early second trimester (14-17 weeks of gestation) in 523 pregnant women. The authors found that AFP alone could predict GDM diagnosis with a sensitivity of 70%, specificity of 93% and AUC of 0.58 (95% CI 0.51-0.62) and AFP combined with unconjugated estriol and β-hCG levels can predict GDM with a sensitivity of 95%, specificity of 86% and AUC of 0.91 (95% CI 0.87-0.98).

Wnt1-inducible signalling pathway protein-1 (WISP1) was studied by Liu *et al.* (332) in 313 pregnant women (GDM n=61, 2h 75g OGTT, IADPSG criteria). The samples were taken at the time of the OGTT. The authors found that WISP1 levels were significantly higher in GDM patients with pre-pregnancy overweight or obesity compare with normoglycemic and normal weight subjects suggesting a possible role of this protein in the mechanisms involved in obesity induced insulin resistance in GDM, hypothesis also suggested by Sahin Esroy *et al.* (333).

Irisin levels were measured by AL-Ghazali *et al.* (334) in 90 pregnant women (GDM n=60, 2h 75g OGTT, IADPSG criteria) at the time of the OGTT and found significantly lower levels of irisin in women with GDM compared to controls. Diagnostic capacity (i.e., AUC) was not calculated. These findings are supported by other studies (335, 336)

Asprosin levels (337) were found to be significantly higher in women with GDM compared with controls at the time of OGTT but also as early as 18-20 weeks of gestation suggesting a potential role as an early biomarker. Similarly spexin and subfatin levels (338) and fibrinogen-like protein 1 (FGL-1) (339) were found to be higher in women with GDM compared with controls (samples taken at the time of the OGTT).

Finally, coiled-coil domain-containing 80 (CCDC80) levels (340) and complement C1q Tumor Necrosis Factor-Related Protein 1 (CTRP1) levels (341) taken at the time of the OGTT (2h 75g OGTT, IADPSG criteria) were significantly lower in women with GDM compare with controls. Additionally, CCDC80 could identify GDM cases with an AUC of 0.61 (95%CI 0.53-0.68) which increased to 0.74 when additional variables were included in the model (maternal age, gestational age, BMI, blood pressure).

Conclusion

This review has identified and described 15 promising biomarkers that could potentially replace the OGTT and be used to both predict and diagnose GDM. Steps required to move the biomarker agenda forward should include large multi-centre, multi-ethnic prospective studies using uniform screening and diagnostic criteria for GDM, with longitudinal sampling in all 3 trimesters and with well recorded patient characteristics. One such study would answer many questions and help identify the best candidate marker. Recently, the Lames Lindt Alliance (Priority Setting Partnerships) has identified the top 10 research priorities for GDM, one of which is the identification of the best test to diagnose GDM (342). The scientific community agrees that the OGTT is a dated, cumbersome, imperfect test and needs to be replaced and this review highlights some very promising contenders.

Funding

This research received no external funding

Conflict of Interest

The authors declare no conflict of interest

Author Contributions

DB, POS and FPD were responsible for the conception and design of the study. DB, CR, DM screened, collected, extracted, and analysed the data. DB drafted the manuscript. All authors (DB, CR, DM, HD, JH, POS, FD) made substantial contributions to the interpretation of data, critically revised the manuscript for important intellectual content and approved the final version to be published. DB is responsible for the integrity of the work as a whole.

References

1. Alberti KG, Zimmet PZ. Definition, diagnosis and classification of diabetes mellitus and its complications. Part 1: diagnosis and classification of diabetes mellitus provisional report of a WHO consultation. Diabet Med. 1998;15(7):539-53.
2. Zhu Y, Zhang C. Prevalence of Gestational Diabetes and Risk of Progression to Type 2 Diabetes: a Global Perspective. Curr Diab Rep. 2016;16(1):7.
3. Goedegebure EAR, Koning SH, Hoogenberg K, Korteweg FJ, Lutgers HL, Diekman MJM, et al. Pregnancy outcomes in women with gestational diabetes mellitus diagnosed according to the WHO-2013 and WHO-1999 diagnostic criteria: a multicentre retrospective cohort study. BMC Pregnancy Childbirth. 2018;18(1):152.
4. Koivunen S, Viljakainen M, Männistö T, Gissler M, Pouta A, Kaaja R, et al. Pregnancy outcomes according to the definition of gestational diabetes. PLoS One. 2020;15(3):e0229496.
5. Metzger BE. Long-term Outcomes in Mothers Diagnosed With Gestational Diabetes Mellitus and Their Offspring. Clinical Obstetrics and Gynecology. 2007;50(4):972-9.
6. O'Sullivan EP, Avalos G, O'Reilly M, Dennedy MC, Gaffney G, Dunne FP, et al. Atlantic DIP: the prevalence and consequences of gestational diabetes in Ireland. Ir Med J. 2012;105(5 Suppl):13-5.
7. Crowther CA, Hiller JE, Moss JR, McPhee AJ, Jeffries WS, Robinson JS, et al. Effect of treatment of gestational diabetes mellitus on pregnancy outcomes. N Engl J Med. 2005;352(24):2477-86.
8. Crowther CA, Hiller JE, Moss JR, McPhee AJ, Jeffries WS, Robinson JS. Effect of Treatment of Gestational Diabetes Mellitus on Pregnancy Outcomes. New England Journal of Medicine. 2005;352(24):2477-86.
9. Landon MB, Spong CY, Thom E, Carpenter MW, Ramin SM, Casey B, et al. A multicenter, randomized trial of treatment for mild gestational diabetes. N Engl J Med. 2009;361(14):1339-48.
10. Koivusalo SB, Rönö K, Klemetti MM, Roine RP, Lindström J, Erkkola M, et al. Gestational Diabetes Mellitus Can Be Prevented by Lifestyle Intervention: The Finnish Gestational Diabetes Prevention Study (RADIEL): A Randomized Controlled Trial. Diabetes Care. 2016;39(1):24-30.
11. International Association of Diabetes and Pregnancy Study Groups Consensus P. International Association of Diabetes and Pregnancy Study Groups Recommendations on the Diagnosis and Classification of Hyperglycemia in Pregnancy. Diabetes Care. 2010;33(3):676-82.
12. Wilkerson HLC, Remein QR. Studies of Abnormal Carbohydrate Metabolism in Pregnancy: The Significance of Impaired Glucose Tolerance. Diabetes. 1957;6(4):324-9.
13. Metzger BE, Gabbe SG, Persson B, Buchanan TA, Catalano PA, Damm P, et al. International association of diabetes and pregnancy study groups recommendations on the diagnosis and classification of hyperglycemia in pregnancy. Diabetes Care. 2010;33(3):676-82.
14. Lachmann EH, Fox RA, Dennison RA, Usher-Smith JA, Meek CL, Aiken CE. Barriers to completing oral glucose tolerance testing in women at risk of gestational diabetes. Diabetic Medicine. 2020;37(9):1482-9.
15. Bogdanet D, O’Shea P, Lyons C, Shafat A, Dunne F. The Oral Glucose Tolerance Test—Is It Time for a Change?—A Literature Review with an Emphasis on Pregnancy. Journal of Clinical Medicine. 2020;9(11):3451.
16. Catalano PM, Avallone DA, Drago NM, Amini SB. Reproducibility of the oral glucose tolerance test in pregnant women. American Journal of Obstetrics and Gynecology. 1993;169(4):874-81.
17. Arksey H, O'Malley L. Scoping studies: towards a methodological framework. International Journal of Social Research Methodology. 2005;8(1):19-32.
18. Levac D, Colquhoun H, O'Brien KK. Scoping studies: advancing the methodology. Implement Sci. 2010;5:69.
19. Peters MD, Godfrey CM, Khalil H, McInerney P, Parker D, Soares CB. Guidance for conducting systematic scoping reviews. Int J Evid Based Healthc. 2015;13(3):141-6.

20. Munn Z, Peters MDJ, Stern C, Tufanaru C, McArthur A, Aromataris E. Systematic review or scoping review? Guidance for authors when choosing between a systematic or scoping review approach. BMC Med Res Methodol. 2018;18(1):143.
21. Tricco AC, Lillie E, Zarin W, O'Brien KK, Colquhoun H, Levac D, et al. PRISMA Extension for Scoping Reviews (PRISMA-ScR): Checklist and Explanation. Ann Intern Med. 2018;169(7):467-73.
22. Ouzzani M, Hammady H, Fedorowicz Z, Elmagarmid A. Rayyan-a web and mobile app for systematic reviews. Syst Rev. 2016;5(1):210.
23. Blüher M. Adipose tissue dysfunction in obesity. Exp Clin Endocrinol Diabetes. 2009;117(6):241-50.
24. Blüher M. Adipokines - removing road blocks to obesity and diabetes therapy. Mol Metab. 2014;3(3):230-40.
25. Maeda K, Okubo K, Shimomura I, Funahashi T, Matsuzawa Y, Matsubara K. cDNA cloning and expression of a novel adipose specific collagen-like factor, apM1 (AdiPose Most abundant Gene transcript 1). Biochem Biophys Res Commun. 1996;221(2):286-9.
26. Martinez-Huenchullan SF, Tam CS, Ban LA, Ehrenfeld-Slater P, Mclennan SV, Twigg SM. Skeletal muscle adiponectin induction in obesity and exercise. Metabolism. 2020;102:154008.
27. Chen J, Tan B, Karteris E, Zervou S, Digby J, Hillhouse EW, et al. Secretion of adiponectin by human placenta: differential modulation of adiponectin and its receptors by cytokines. Diabetologia. 2006;49(6):1292-302.
28. Yamauchi T, Kamon J, Waki H, Terauchi Y, Kubota N, Hara K, et al. The fat-derived hormone adiponectin reverses insulin resistance associated with both lipoatrophy and obesity. Nat Med. 2001;7(8):941-6.
29. Berg AH, Combs TP, Du X, Brownlee M, Scherer PE. The adipocyte-secreted protein Acrp30 enhances hepatic insulin action. Nat Med. 2001;7(8):947-53.
30. Combs TP, Berg AH, Obici S, Scherer PE, Rossetti L. Endogenous glucose production is inhibited by the adipose-derived protein Acrp30. J Clin Invest. 2001;108(12):1875-81.
31. Fruebis J, Tsao TS, Javorschi S, Ebbets-Reed D, Erickson MR, Yen FT, et al. Proteolytic cleavage product of 30-kDa adipocyte complement-related protein increases fatty acid oxidation in muscle and causes weight loss in mice. Proc Natl Acad Sci U S A. 2001;98(4):2005-10.
32. Duncan BB, Schmidt MI, Pankow JS, Bang H, Couper D, Ballantyne CM, et al. Adiponectin and the development of type 2 diabetes: the atherosclerosis risk in communities study. Diabetes. 2004;53(9):2473-8.
33. Wang Y, Meng RW, Kunutsor SK, Chowdhury R, Yuan JM, Koh WP, et al. Plasma adiponectin levels and type 2 diabetes risk: a nested case-control study in a Chinese population and an updated meta-analysis. Sci Rep. 2018;8(1):406.
34. Mohammadi T, Paknahad Z. Adiponectin Concentration in Gestational Diabetic Women: a Case-Control Study. Clin Nutr Res. 2017;6(4):267-76.
35. Ranheim T, Haugen F, Staff AC, Braekke K, Harsem NK, Drevon CA. Adiponectin is reduced in gestational diabetes mellitus in normal weight women. Acta Obstet Gynecol Scand. 2004;83(4):341-7.
36. Hedderson MM, Darbinian J, Havel PJ, Quesenberry CP, Sridhar S, Ehrlich S, et al. Low prepregnancy adiponectin concentrations are associated with a marked increase in risk for development of gestational diabetes mellitus. Diabetes Care. 2013;36(12):3930-7.
37. Committee opinion no. 504: Screening and diagnosis of gestational diabetes mellitus. Obstet Gynecol. 2011;118(3):751-3.
38. Georgiou HM, Lappas M, Georgiou GM, Marita A, Bryant VJ, Hiscock R, et al. Screening for biomarkers predictive of gestational diabetes mellitus. Acta Diabetol. 2008;45(3):157-65.
39. Williams MA, Qiu C, Muy-Rivera M, Vadachkoria S, Song T, Luthy DA. Plasma adiponectin concentrations in early pregnancy and subsequent risk of gestational diabetes mellitus. J Clin Endocrinol Metab. 2004;89(5):2306-11.

40. Ferreira AF, Rezende JC, Vaikousi E, Akolekar R, Nicolaides KH. Maternal serum visfatin at 11-13 weeks of gestation in gestational diabetes mellitus. Clin Chem. 2011;57(4):609-13.
41. Madhu SV, Bhardwaj S, Jhamb R, Srivastava H, Sharma S, Raizada N. Prediction of Gestational Diabetes from First Trimester Serum Adiponectin Levels in Indian Women. Indian J Endocrinol Metab. 2019;23(5):536-9.
42. Bozkurt L, Göbl CS, Baumgartner-Parzer S, Luger A, Pacini G, Kautzky-Willer A. Adiponectin and Leptin at Early Pregnancy: Association to Actual Glucose Disposal and Risk for GDM-A Prospective Cohort Study. Int J Endocrinol. 2018;2018:5463762.
43. Aguilar-Salinas CA, García EG, Robles L, Riaño D, Ruiz-Gomez DG, García-Ulloa AC, et al. High adiponectin concentrations are associated with the metabolically healthy obese phenotype. J Clin Endocrinol Metab. 2008;93(10):4075-9.
44. Ahl S, Guenther M, Zhao S, James R, Marks J, Szabo A, et al. Adiponectin Levels Differentiate Metabolically Healthy vs Unhealthy Among Obese and Nonobese White Individuals. J Clin Endocrinol Metab. 2015;100(11):4172-80.
45. Weerakiet S, Lertnarkorn K, Panburana P, Pitakitronakorn S, Vesathada K, Wansumrith S. Can adiponectin predict gestational diabetes? Gynecol Endocrinol. 2006;22(7):362-8.
46. Xu J, Zhao YH, Chen YP, Yuan XL, Wang J, Zhu H, et al. Maternal circulating concentrations of tumor necrosis factor-alpha, leptin, and adiponectin in gestational diabetes mellitus: a systematic review and meta-analysis. ScientificWorldJournal. 2014;2014:926932.
47. Iliodromiti S, Sassarini J, Kelsey TW, Lindsay RS, Sattar N, Nelson SM. Accuracy of circulating adiponectin for predicting gestational diabetes: a systematic review and meta-analysis. Diabetologia. 2016;59(4):692-9.
48. Sattar N, Wannamethee SG, Forouhi NG. Novel biochemical risk factors for type 2 diabetes: pathogenic insights or prediction possibilities? Diabetologia. 2008;51(6):926-40.
49. Yeral MI, Ozgu-Erdinc AS, Uygur D, Seckin KD, Karsli MF, Danisman AN. Prediction of gestational diabetes mellitus in the first trimester, comparison of fasting plasma glucose, two-step and one-step methods: a prospective randomized controlled trial. Endocrine. 2014;46(3):512-8.
50. Trujillo J, Vigo A, Reichelt A, Duncan BB, Schmidt MI. Fasting plasma glucose to avoid a full OGTT in the diagnosis of gestational diabetes. Diabetes Res Clin Pract. 2014;105(3):322-6.
51. Thewjitcharoen Y, Jones Elizabeth A, Butadej S, Nakasatien S, Chotwanvirat P, Wanothayaroj E, et al. Performance of HbA1c versus oral glucose tolerance test (OGTT) as a screening tool to diagnose dysglycemic status in high-risk Thai patients. BMC Endocr Disord. 2019;19(1):23.
52. Aziz NL, Abdelwahab S, Moussa M, Georgy M. Maternal fructosamine and glycosylated haemoglobin in the prediction of gestational glucose intolerance. Clin Exp Obstet Gynecol. 1992;19(4):235-41.
53. Nagpal S, Patel S, Jacobe H, DiSepio D, Ghosn C, Malhotra M, et al. Tazarotene-induced gene 2 (TIG2), a novel retinoid-responsive gene in skin. J Invest Dermatol. 1997;109(1):91-5.
54. Goralski KB, McCarthy TC, Hanniman EA, Zabel BA, Butcher EC, Parlee SD, et al. Chemerin, a novel adipokine that regulates adipogenesis and adipocyte metabolism. J Biol Chem. 2007;282(38):28175-88.
55. Sell H, Laurencikiene J, Taube A, Eckardt K, Cramer A, Horrighs A, et al. Chemerin is a novel adipocyte-derived factor inducing insulin resistance in primary human skeletal muscle cells. Diabetes. 2009;58(12):2731-40.
56. Huang J, Zhang J, Lei T, Chen X, Zhang Y, Zhou L, et al. Cloning of porcine chemerin, ChemR23 and GPR1 and their involvement in regulation of lipogenesis. BMB Rep. 2010;43(7):491-8.
57. Garces MF, Sanchez E, Acosta BJ, Angel E, Ruíz AI, Rubio-Romero JA, et al. Expression and regulation of chemerin during rat pregnancy. Placenta. 2012;33(5):373-8.
58. Bozaoglu K, Bolton K, McMillan J, Zimmet P, Jowett J, Collier G, et al. Chemerin is a novel adipokine associated with obesity and metabolic syndrome. Endocrinology. 2007;148(10):4687-94.

59. Stepan H, Philipp A, Roth I, Kralisch S, Jank A, Schaarschmidt W, et al. Serum levels of the adipokine chemerin are increased in preeclampsia during and 6 months after pregnancy. Regul Pept. 2011;168(1-3):69-72.
60. Duan DM, Niu JM, Lei Q, Lin XH, Chen X. Serum levels of the adipokine chemerin in preeclampsia. J Perinat Med. 2011;40(2):121-7.
61. Xu QL, Zhu M, Jin Y, Wang N, Xu HX, Quan LM, et al. The predictive value of the first-trimester maternal serum chemerin level for pre-eclampsia. Peptides. 2014;62:150-4.
62. Yang X, Quan X, Lan Y, Ye J, Wei Q, Yin X, et al. Serum chemerin level during the first trimester of pregnancy and the risk of gestational diabetes mellitus. Gynecol Endocrinol. 2017;33(10):770-3.
63. Wang X, Liu J, Wang D, Zhu H, Kang L, Jiang J. Expression and correlation of Chemerin and FABP4 in peripheral blood of gestational diabetes mellitus patients. Exp Ther Med. 2020;19(1):710-6.
64. Pfau D, Stepan H, Kratzsch J, Verlohren M, Verlohren HJ, Drynda K, et al. Circulating levels of the adipokine chemerin in gestational diabetes mellitus. Horm Res Paediatr. 2010;74(1):56-61.
65. Guelfi KJ, Ong MJ, Li S, Wallman KE, Doherty DA, Fournier PA, et al. Maternal circulating adipokine profile and insulin resistance in women at high risk of developing gestational diabetes mellitus. Metabolism. 2017;75:54-60.
66. van Poppel MN, Zeck W, Ulrich D, Schest EC, Hirschmugl B, Lang U, et al. Cord blood chemerin: differential effects of gestational diabetes mellitus and maternal obesity. Clin Endocrinol (Oxf). 2014;80(1):65-72.
67. Sun J, Ren J, Zuo C, Deng D, Pan F, Chen R, et al. Circulating apelin, chemerin and omentin levels in patients with gestational diabetes mellitus: a systematic review and meta-analysis. Lipids Health Dis. 2020;19(1):26.
68. Zhou Z, Chen H, Ju H, Sun M. Circulating chemerin levels and gestational diabetes mellitus: a systematic review and meta-analysis. Lipids Health Dis. 2018;17(1):169.
69. El-Mesallamy HO, El-Derany MO, Hamdy NM. Serum omentin-1 and chemerin levels are interrelated in patients with Type 2 diabetes mellitus with or without ischaemic heart disease. Diabet Med. 2011;28(10):1194-200.
70. Sell H, Divoux A, Poitou C, Basdevant A, Bouillot JL, Bedossa P, et al. Chemerin correlates with markers for fatty liver in morbidly obese patients and strongly decreases after weight loss induced by bariatric surgery. J Clin Endocrinol Metab. 2010;95(6):2892-6.
71. Kukla M, Zwirska-Korczala K, Hartleb M, Waluga M, Chwist A, Kajor M, et al. Serum chemerin and vaspin in non-alcoholic fatty liver disease. Scand J Gastroenterol. 2010;45(2):235-42.
72. Chakaroun R, Raschpichler M, Klöting N, Oberbach A, Flehmig G, Kern M, et al. Effects of weight loss and exercise on chemerin serum concentrations and adipose tissue expression in human obesity. Metabolism. 2012;61(5):706-14.
73. Weigert J, Neumeier M, Wanninger J, Filarsky M, Bauer S, Wiest R, et al. Systemic chemerin is related to inflammation rather than obesity in type 2 diabetes. Clin Endocrinol (Oxf). 2010;72(3):342-8.
74. Brix JM, Stingl H, Höllerl F, Schernthaner GH, Kopp HP, Schernthaner G. Elevated Fetuin-A concentrations in morbid obesity decrease after dramatic weight loss. J Clin Endocrinol Metab. 2010;95(11):4877-81.
75. Trepanowski JF, Mey J, Varady KA. Fetuin-A: a novel link between obesity and related complications. Int J Obes (Lond). 2015;39(5):734-41.
76. Ix JH, Shlipak MG, Brandenburg VM, Ali S, Ketteler M, Whooley MA. Association between human fetuin-A and the metabolic syndrome: data from the Heart and Soul Study. Circulation. 2006;113(14):1760-7.
77. Ou HY, Yang YC, Wu HT, Wu JS, Lu FH, Chang CJ. Increased fetuin-A concentrations in impaired glucose tolerance with or without nonalcoholic fatty liver disease, but not impaired fasting glucose. J Clin Endocrinol Metab. 2012;97(12):4717-23.

78. Jensen MK, Bartz TM, Mukamal KJ, Djoussé L, Kizer JR, Tracy RP, et al. Fetuin-A, type 2 diabetes, and risk of cardiovascular disease in older adults: the cardiovascular health study. Diabetes Care. 2013;36(5):1222-8.
79. Ou HY, Yang YC, Wu HT, Wu JS, Lu FH, Chang CJ. Serum fetuin-A concentrations are elevated in subjects with impaired glucose tolerance and newly diagnosed type 2 diabetes. Clin Endocrinol (Oxf). 2011;75(4):450-5.
80. Denecke B, Gräber S, Schäfer C, Heiss A, Wöltje M, Jahnen-Dechent W. Tissue distribution and activity testing suggest a similar but not identical function of fetuin-B and fetuin-A. Biochem J. 2003;376(Pt 1):135-45.
81. Meex RC, Hoy AJ, Morris A, Brown RD, Lo JC, Burke M, et al. Fetuin B Is a Secreted Hepatocyte Factor Linking Steatosis to Impaired Glucose Metabolism. Cell Metab. 2015;22(6):1078-89.
82. Peter A, Kovarova M, Staiger H, Machann J, Schick F, Königsrainer A, et al. The hepatokines fetuin-A and fetuin-B are upregulated in the state of hepatic steatosis and may differently impact on glucose homeostasis in humans. Am J Physiol Endocrinol Metab. 2018;314(3):E266-E73.
83. Kansu-Celik H, Ozgu-Erdinc AS, Kisa B, Findik RB, Yilmaz C, Tasci Y. Prediction of gestational diabetes mellitus in the first trimester: comparison of maternal fetuin-A, N-terminal proatrial natriuretic peptide, high-sensitivity C-reactive protein, and fasting glucose levels. Archives of Endocrinology and Metabolism. 2019.
84. Kalabay L, Cseh K, Pajor A, Baranyi E, Csákány GM, Melczer Z, et al. Correlation of maternal serum fetuin/alpha2-HS-glycoprotein concentration with maternal insulin resistance and anthropometric parameters of neonates in normal pregnancy and gestational diabetes. Eur J Endocrinol. 2002;147(2):243-8.
85. Iyidir OT, Degertekin CK, Yilmaz BA, Altinova AE, Toruner FB, Bozkurt N, et al. Serum levels of fetuin A are increased in women with gestational diabetes mellitus. Arch Gynecol Obstet. 2015;291(4):933-7.
86. Coustan DR, Carpenter MW. The diagnosis of gestational diabetes. Diabetes Care. 1998;21 Suppl 2:B5-8.
87. Jin C, Lin L, Han N, Zhao Z, Liu Z, Luo S, et al. Effects of dynamic change in fetuin-A levels from the first to the second trimester on insulin resistance and gestational diabetes mellitus: a nested case-control study. BMJ Open Diabetes Res Care. 2020;8(1).
88. Farhan S, Handisurya A, Todoric J, Tura A, Pacini G, Wagner O, et al. Fetuin-A Characteristics during and after Pregnancy: Result from a Case Control Pilot Study. Int J Endocrinol. 2012;2012:896736.
89. Zhang Y, Proenca R, Maffei M, Barone M, Leopold L, Friedman JM. Positional cloning of the mouse obese gene and its human homologue. Nature. 1994;372(6505):425-32.
90. Considine RV, Sinha MK, Heiman ML, Kriauciunas A, Stephens TW, Nyce MR, et al. Serum immunoreactive-leptin concentrations in normal-weight and obese humans. N Engl J Med. 1996;334(5):292-5.
91. Bado A, Levasseur S, Attoub S, Kermorgant S, Laigneau JP, Bortoluzzi MN, et al. The stomach is a source of leptin. Nature. 1998;394(6695):790-3.
92. Masuzaki H, Ogawa Y, Sagawa N, Hosoda K, Matsumoto T, Mise H, et al. Nonadipose tissue production of leptin: leptin as a novel placenta-derived hormone in humans. Nat Med. 1997;3(9):1029-33.
93. Wilkinson M, Morash B, Ur E. The brain is a source of leptin. Front Horm Res. 2000;26:106-25.
94. Considine RV. Human leptin: an adipocyte hormone with weight-regulatory and endocrine functions. Semin Vasc Med. 2005;5(1):15-24.
95. Brennan AM, Mantzoros CS. Drug Insight: the role of leptin in human physiology and pathophysiology--emerging clinical applications. Nat Clin Pract Endocrinol Metab. 2006;2(6):318-27.

96. Robertson SA, Leinninger GM, Myers MG. Molecular and neural mediators of leptin action. Physiol Behav. 2008;94(5):637-42.
97. Harris RB, Zhou J, Redmann SM, Smagin GN, Smith SR, Rodgers E, et al. A leptin dose-response study in obese (ob/ob) and lean (+/?) mice. Endocrinology. 1998;139(1):8-19.
98. Gavrilova O, Marcus-Samuels B, Graham D, Kim JK, Shulman GI, Castle AL, et al. Surgical implantation of adipose tissue reverses diabetes in lipoatrophic mice. J Clin Invest. 2000;105(3):271-8.
99. Farooqi IS, Matarese G, Lord GM, Keogh JM, Lawrence E, Agwu C, et al. Beneficial effects of leptin on obesity, T cell hyporesponsiveness, and neuroendocrine/metabolic dysfunction of human congenital leptin deficiency. J Clin Invest. 2002;110(8):1093-103.
100. Lewandowski K, Horn R, O'Callaghan CJ, Dunlop D, Medley GF, O'Hare P, et al. Free leptin, bound leptin, and soluble leptin receptor in normal and diabetic pregnancies. J Clin Endocrinol Metab. 1999;84(1):300-6.
101. Bawah AT, Seini MM, Abaka-Yawason A, Alidu H, Nanga S. Leptin, resistin and visfatin as useful predictors of gestational diabetes mellitus. Lipids Health Dis. 2019;18(1):221.
102. Association AD. Diagnosis and classification of diabetes mellitus. Diabetes Care. 2010;33 Suppl 1:S62-9.
103. Kautzky-Willer A, Pacini G, Tura A, Bieglmayer C, Schneider B, Ludvik B, et al. Increased plasma leptin in gestational diabetes. Diabetologia. 2001;44(2):164-72.
104. Alberti KG, Zimmet PZ. Definition, diagnosis and classification of diabetes mellitus and its complications. Part 1: diagnosis and classification of diabetes mellitus provisional report of a WHO consultation. Diabetic Medicine: A Journal of the British Diabetic Association. 1998;15(7):539-53.
105. Boyadzhieva M, Atanasova I, Zacharieva S, Kedikova S. Adipocytokines during pregnancy and postpartum in women with gestational diabetes and healthy controls. J Endocrinol Invest. 2013;36(11):944-9.
106. Bao W, Baecker A, Song Y, Kiely M, Liu S, Zhang C. Adipokine levels during the first or early second trimester of pregnancy and subsequent risk of gestational diabetes mellitus: A systematic review. Metabolism. 2015;64(6):756-64.
107. McLachlan KA, O'Neal D, Jenkins A, Alford FP. Do adiponectin, TNFalpha, leptin and CRP relate to insulin resistance in pregnancy? Studies in women with and without gestational diabetes, during and after pregnancy. Diabetes Metab Res Rev. 2006;22(2):131-8.
108. Olefsky JM, Farquhar JW, Reaven GM. Do the oral and intravenous glucose tolerance tests provide similar diagnostic information in patients with chemical diabetes mellitus? Diabetes. 1973;22(3):202-9.
109. Otsuka R, Yatsuya H, Tamakoshi K, Matsushita K, Wada K, Toyoshima H. Perceived psychological stress and serum leptin concentrations in Japanese men. Obesity (Silver Spring). 2006;14(10):1832-8.
110. Knutson KL, Spiegel K, Penev P, Van Cauter E. The metabolic consequences of sleep deprivation. Sleep Med Rev. 2007;11(3):163-78.
111. de Salles BF, Simão R, Fleck SJ, Dias I, Kraemer-Aguiar LG, Bouskela E. Effects of resistance training on cytokines. Int J Sports Med. 2010;31(7):441-50.
112. Yang RZ, Lee MJ, Hu H, Pray J, Wu HB, Hansen BC, et al. Identification of omentin as a novel depot-specific adipokine in human adipose tissue: possible role in modulating insulin action. Am J Physiol Endocrinol Metab. 2006;290(6):E1253-61.
113. Schäffler A, Neumeier M, Herfarth H, Fürst A, Schölmerich J, Büchler C. Genomic structure of human omentin, a new adipocytokine expressed in omental adipose tissue. Biochim Biophys Acta. 2005;1732(1-3):96-102.
114. de Souza Batista CM, Yang RZ, Lee MJ, Glynn NM, Yu DZ, Pray J, et al. Omentin plasma levels and gene expression are decreased in obesity. Diabetes. 2007;56(6):1655-61.
115. Barker G, Lim R, Georgiou HM, Lappas M. Omentin-1 is decreased in maternal plasma, placenta and adipose tissue of women with pre-existing obesity. PLoS One. 2012;7(8):e42943.

116. Hoffman L, Nolan C, Wilson JD, Oats JJ, Simmons D. Gestational diabetes mellitus--management guidelines. The Australasian Diabetes in Pregnancy Society. Med J Aust. 1998;169(2):93-7.
117. Abell SK, Shorakae S, Harrison CL, Hiam D, Moreno-Asso A, Stepto NK, et al. The association between dysregulated adipocytokines in early pregnancy and development of gestational diabetes. Diabetes Metab Res Rev. 2017;33(8).
118. Franz M, Polterauer M, Springer S, Kuessel L, Haslinger P, Worda C, et al. Maternal and neonatal omentin-1 levels in gestational diabetes. Arch Gynecol Obstet. 2018;297(4):885-9.
119. Metzger BE, Lowe LP, Dyer AR, Trimble ER, Chaovarindr U, Coustan DR, et al. Hyperglycemia and adverse pregnancy outcomes. N Engl J Med. 2008;358(19):1991-2002.
120. Ebrahimi S, Gargari BP, Izadi A, Imani B, Asjodi F. The effects of Ramadan fasting on serum concentrations of vaspin and omentin-1 in patients with nonalcoholic fatty liver disease. European Journal of Integrative Medicine. 2018;19:110-4.
121. Kiyak Caglayan E, Engin-Ustun Y, Sari N, Gocmen AY, Polat MF. The effects of prolonged fasting on the levels of adiponectin, leptin, apelin, and omentin in pregnant women. J Obstet Gynaecol. 2016;36(4):555-8.
122. Kamimura D, Ishihara K, Hirano T. IL-6 signal transduction and its physiological roles: the signal orchestration model. Rev Physiol Biochem Pharmacol. 2003;149:1-38.
123. Van Snick J. Interleukin-6: an overview. Annu Rev Immunol. 1990;8:253-78.
124. Jordan SC, Choi J, Kim I, Wu G, Toyoda M, Shin B, et al. Interleukin-6, A Cytokine Critical to Mediation of Inflammation, Autoimmunity and Allograft Rejection: Therapeutic Implications of IL-6 Receptor Blockade. Transplantation. 2017;101(1):32-44.
125. Senn JJ, Klover PJ, Nowak IA, Mooney RA. Interleukin-6 induces cellular insulin resistance in hepatocytes. Diabetes. 2002;51(12):3391-9.
126. Kim JH, Bachmann RA, Chen J. Interleukin-6 and insulin resistance. Vitam Horm. 2009;80:613-33.
127. Hoene M, Weigert C. The role of interleukin-6 in insulin resistance, body fat distribution and energy balance. Obes Rev. 2008;9(1):20-9.
128. Carey AL, Bruce CR, Sacchetti M, Anderson MJ, Olsen DB, Saltin B, et al. Interleukin-6 and tumor necrosis factor-alpha are not increased in patients with Type 2 diabetes: evidence that plasma interleukin-6 is related to fat mass and not insulin responsiveness. Diabetologia. 2004;47(6):1029-37.
129. Dekker MJ, Lee S, Hudson R, Kilpatrick K, Graham TE, Ross R, et al. An exercise intervention without weight loss decreases circulating interleukin-6 in lean and obese men with and without type 2 diabetes mellitus. Metabolism. 2007;56(3):332-8.
130. Suzuki T, Imai J, Yamada T, Ishigaki Y, Kaneko K, Uno K, et al. Interleukin-6 enhances glucose-stimulated insulin secretion from pancreatic beta-cells: potential involvement of the PLC-IP3-dependent pathway. Diabetes. 2011;60(2):537-47.
131. Trujillo ME, Sullivan S, Harten I, Schneider SH, Greenberg AS, Fried SK. Interleukin-6 regulates human adipose tissue lipid metabolism and leptin production in vitro. J Clin Endocrinol Metab. 2004;89(11):5577-82.
132. Wallenius K, Wallenius V, Sunter D, Dickson SL, Jansson JO. Intracerebroventricular interleukin-6 treatment decreases body fat in rats. Biochem Biophys Res Commun. 2002;293(1):560-5.
133. Stenlöf K, Wernstedt I, Fjällman T, Wallenius V, Wallenius K, Jansson JO. Interleukin-6 levels in the central nervous system are negatively correlated with fat mass in overweight/obese subjects. J Clin Endocrinol Metab. 2003;88(9):4379-83.
134. Sudharshana Murthy KA, Bhandiwada A, Chandan SL, Gowda SL, Sindhusree G. Evaluation of Oxidative Stress and Proinflammatory Cytokines in Gestational Diabetes Mellitus and Their Correlation with Pregnancy Outcome. Indian J Endocrinol Metab. 2018;22(1):79-84.

135. Siddiqui S, Waghdhare S, Goel C, Panda M, Soneja H, Sundar J, et al. Augmentation of IL-6 production contributes to development of gestational diabetes mellitus: An Indian study. Diabetes Metab Syndr. 2019;13(2):895-9.
136. Braga FO, Negrato CA, Matta MFBD, Carneiro JRI, Gomes MB. Relationship between inflammatory markers, glycated hemoglobin and placental weight on fetal outcomes in women with gestational diabetes. Arch Endocrinol Metab. 2019;63(1):22-9.
137. Šimják P, Cinkajzlová A, Anderlová K, Kloučková J, Kratochvílová H, Lacinová Z, et al. Changes in plasma concentrations and mRNA expression of hepatokines fetuin A, fetuin B and FGF21 in physiological pregnancy and gestational diabetes mellitus. Physiol Res. 2018;67(Suppl 3):S531-S42.
138. Amirian A, Mahani MB, Abdi F. Role of interleukin-6 (IL-6) in predicting gestational diabetes mellitus. Obstet Gynecol Sci. 2020;63(4):407-16.
139. Abdel Gader AG, Khashoggi TY, Habib F, Awadallah SB. Haemostatic and cytokine changes in gestational diabetes mellitus. Gynecol Endocrinol. 2011;27(5):356-60.
140. Wolf M, Sauk J, Shah A, Vossen Smirnakis K, Jimenez-Kimble R, Ecker JL, et al. Inflammation and glucose intolerance: a prospective study of gestational diabetes mellitus. Diabetes Care. 2004;27(1):21-7.
141. Wedell-Neergaard AS, Lang Lehrskov L, Christensen RH, Legaard GE, Dorph E, Larsen MK, et al. Exercise-Induced Changes in Visceral Adipose Tissue Mass Are Regulated by IL-6 Signaling: A Randomized Controlled Trial. Cell Metab. 2019;29(4):844-55.e3.
142. Desoye G, Hauguel-de Mouzon S. The human placenta in gestational diabetes mellitus. The insulin and cytokine network. Diabetes Care. 2007;30 Suppl 2:S120-6.
143. Grivennikov SI, Tumanov AV, Liepinsh DJ, Kruglov AA, Marakusha BI, Shakhov AN, et al. Distinct and nonredundant in vivo functions of TNF produced by t cells and macrophages/neutrophils: protective and deleterious effects. Immunity. 2005;22(1):93-104.
144. Chen HL, Yang YP, Hu XL, Yelavarthi KK, Fishback JL, Hunt JS. Tumor necrosis factor alpha mRNA and protein are present in human placental and uterine cells at early and late stages of gestation. Am J Pathol. 1991;139(2):327-35.
145. Carswell EA, Old LJ, Kassel RL, Green S, Fiore N, Williamson B. An endotoxin-induced serum factor that causes necrosis of tumors. Proc Natl Acad Sci U S A. 1975;72(9):3666-70.
146. Williams RO, Paleolog E, Feldmann M. Cytokine inhibitors in rheumatoid arthritis and other autoimmune diseases. Curr Opin Pharmacol. 2007;7(4):412-7.
147. Tweedie D, Sambamurti K, Greig NH. TNF-alpha inhibition as a treatment strategy for neurodegenerative disorders: new drug candidates and targets. Curr Alzheimer Res. 2007;4(4):378-85.
148. Bortolato B, Carvalho AF, Soczynska JK, Perini GI, McIntyre RS. The Involvement of TNF-α in Cognitive Dysfunction Associated with Major Depressive Disorder: An Opportunity for Domain Specific Treatments. Curr Neuropharmacol. 2015;13(5):558-76.
149. Mohammadi M, Gozashti MH, Aghadavood M, Mehdizadeh MR, Hayatbakhsh MM. Clinical Significance of Serum IL-6 and TNF-α Levels in Patients with Metabolic Syndrome. Rep Biochem Mol Biol. 2017;6(1):74-9.
150. Emanuela F, Grazia M, Marco dR, Maria Paola L, Giorgio F, Marco B. Inflammation as a Link between Obesity and Metabolic Syndrome. J Nutr Metab. 2012;2012:476380.
151. Bastard JP, Maachi M, Lagathu C, Kim MJ, Caron M, Vidal H, et al. Recent advances in the relationship between obesity, inflammation, and insulin resistance. Eur Cytokine Netw. 2006;17(1):4-12.
152. Lorenzo M, Fernández-Veledo S, Vila-Bedmar R, Garcia-Guerra L, De Alvaro C, Nieto-Vazquez I. Insulin resistance induced by tumor necrosis factor-alpha in myocytes and brown adipocytes. J Anim Sci. 2008;86(14 Suppl):E94-104.
153. Nieto-Vazquez I, Fernández-Veledo S, Krämer DK, Vila-Bedmar R, Garcia-Guerra L, Lorenzo M. Insulin resistance associated to obesity: the link TNF-alpha. Arch Physiol Biochem. 2008;114(3):183-94.

154. Wang B, Trayhurn P. Acute and prolonged effects of TNF-alpha on the expression and secretion of inflammation-related adipokines by human adipocytes differentiated in culture. Pflugers Arch. 2006;452(4):418-27.
155. Patel AB, Tsilioni I, Weng Z, Theoharides TC. TNF stimulates IL-6, CXCL8 and VEGF secretion from human keratinocytes via activation of mTOR, inhibited by tetramethoxyluteolin. Exp Dermatol. 2018;27(2):135-43.
156. Chang E, Choi JM, Kim WJ, Rhee EJ, Oh KW, Lee WY, et al. Restoration of adiponectin expression via the ERK pathway in TNFα-treated 3T3-L1 adipocytes. Mol Med Rep. 2014;10(2):905-10.
157. Prins JB, Niesler CU, Winterford CM, Bright NA, Siddle K, O'Rahilly S, et al. Tumor necrosis factor-alpha induces apoptosis of human adipose cells. Diabetes. 1997;46(12):1939-44.
158. Nisoli E, Briscini L, Giordano A, Tonello C, Wiesbrock SM, Uysal KT, et al. Tumor necrosis factor alpha mediates apoptosis of brown adipocytes and defective brown adipocyte function in obesity. Proc Natl Acad Sci U S A. 2000;97(14):8033-8.
159. Hotamisligil GS, Peraldi P, Budavari A, Ellis R, White MF, Spiegelman BM. IRS-1-mediated inhibition of insulin receptor tyrosine kinase activity in TNF-alpha- and obesity-induced insulin resistance. Science. 1996;271(5249):665-8.
160. Alzamil H. Elevated Serum TNF-<i>α</i> Is Related to Obesity in Type 2 Diabetes Mellitus and Is Associated with Glycemic Control and Insulin Resistance. Journal of Obesity. 2020;2020:5076858.
161. Guillemette L, Lacroix M, Battista MC, Doyon M, Moreau J, Ménard J, et al. TNFα dynamics during the oral glucose tolerance test vary according to the level of insulin resistance in pregnant women. J Clin Endocrinol Metab. 2014;99(5):1862-9.
162. Kirwan JP, Hauguel-De Mouzon S, Lepercq J, Challier JC, Huston-Presley L, Friedman JE, et al. TNF-alpha is a predictor of insulin resistance in human pregnancy. Diabetes. 2002;51(7):2207-13.
163. Syngelaki A, Visser GH, Krithinakis K, Wright A, Nicolaides KH. First trimester screening for gestational diabetes mellitus by maternal factors and markers of inflammation. Metabolism. 2016;65(3):131-7.
164. Mushtaq R, Akram A, Khwaja S, Ahmed S. The role of inflammatory markers following Ramadan Fasting. Pak J Med Sci. 2019;35(1):77-81.
165. Shojaie M, Ghanbari F, Shojaie N. Intermittent fasting could ameliorate cognitive function against distress by regulation of inflammatory response pathway. J Adv Res. 2017;8(6):697-701.
166. Chandrashekara S, Jayashree K, Veeranna HB, Vadiraj HS, Ramesh MN, Shobha A, et al. Effects of anxiety on TNF-alpha levels during psychological stress. J Psychosom Res. 2007;63(1):65-9.
167. Stewart LK, Flynn MG, Campbell WW, Craig BA, Robinson JP, Timmerman KL, et al. The influence of exercise training on inflammatory cytokines and C-reactive protein. Med Sci Sports Exerc. 2007;39(10):1714-9.
168. Paolucci EM, Loukov D, Bowdish DME, Heisz JJ. Exercise reduces depression and inflammation but intensity matters. Biol Psychol. 2018;133:79-84.
169. Jerkovic L, Voegele AF, Chwatal S, Kronenberg F, Radcliffe CM, Wormald MR, et al. Afamin is a novel human vitamin E-binding glycoprotein characterization and in vitro expression. J Proteome Res. 2005;4(3):889-99.
170. Dieplinger B, Egger M, Gabriel C, Poelz W, Morandell E, Seeber B, et al. Analytical characterization and clinical evaluation of an enzyme-linked immunosorbent assay for measurement of afamin in human plasma. Clin Chim Acta. 2013;425:236-41.
171. Voegele AF, Jerković L, Wellenzohn B, Eller P, Kronenberg F, Liedl KR, et al. Characterization of the vitamin E-binding properties of human plasma afamin. Biochemistry. 2002;41(49):14532-8.
172. Köninger A, Edimiris P, Koch L, Enekwe A, Lamina C, Kasimir-Bauer S, et al. Serum concentrations of afamin are elevated in patients with polycystic ovary syndrome. Endocr Connect. 2014;3(3):120-6.

173. Seeber B, Morandell E, Lunger F, Wildt L, Dieplinger H. Afamin serum concentrations are associated with insulin resistance and metabolic syndrome in polycystic ovary syndrome. Reprod Biol Endocrinol. 2014;12:88.
174. Kollerits B, Lamina C, Huth C, Marques-Vidal P, Kiechl S, Seppälä I, et al. Plasma Concentrations of Afamin Are Associated With Prevalent and Incident Type 2 Diabetes: A Pooled Analysis in More Than 20,000 Individuals. Diabetes Care. 2017;40(10):1386-93.
175. Hubalek M, Buchner H, Mörtl MG, Schlembach D, Huppertz B, Firulovic B, et al. The vitamin E-binding protein afamin increases in maternal serum during pregnancy. Clin Chim Acta. 2014;434:41-7.
176. Tramontana A, Dieplinger B, Stangl G, Hafner E, Dieplinger H. First trimester serum afamin concentrations are associated with the development of pre-eclampsia and gestational diabetes mellitus in pregnant women. Clin Chim Acta. 2018;476:160-6.
177. Tramontana A, Pablik E, Stangl G, Hartmann B, Dieplinger H, Hafner E. Combination of first trimester serum afamin levels and three-dimensional placental bed vascularization as a possible screening method to detect women at-risk for adverse pregnancy complications like pre-eclampsia and gestational diabetes mellitus in low-risk pregnancies. Placenta. 2018;62:9-15.
178. Köninger A, Iannaccone A, Hajder E, Frank M, Schmidt B, Schleussner E, et al. Afamin predicts gestational diabetes in polycystic ovary syndrome patients preconceptionally. Endocr Connect. 2019;8(5):616-24.
179. Ravnsborg T, Svaneklink S, Andersen LLT, Larsen MR, Jensen DM, Overgaard M. First-trimester proteomic profiling identifies novel predictors of gestational diabetes mellitus. PLoS One. 2019;14(3):e0214457.
180. Jensen DM, Mølsted-Pedersen L, Beck-Nielsen H, Westergaard JG, Ovesen P, Damm P. Screening for gestational diabetes mellitus by a model based on risk indicators: a prospective study. Am J Obstet Gynecol. 2003;189(5):1383-8.
181. Köninger A, Mathan A, Mach P, Frank M, Schmidt B, Schleussner E, et al. Is Afamin a novel biomarker for gestational diabetes mellitus? A pilot study. Reprod Biol Endocrinol. 2018;16(1):30.
182. Kleinwechter H, Schäfer-Graf U, Bührer C, Hoesli I, Kainer F, Kautzky-Willer A, et al. Gestational diabetes mellitus (GDM) diagnosis, therapy and follow-up care: Practice Guideline of the German Diabetes Association(DDG) and the German Association for Gynaecologyand Obstetrics (DGGG). Exp Clin Endocrinol Diabetes. 2014;122(7):395-405.
183. Dieplinger H, Dieplinger B. Afamin--A pleiotropic glycoprotein involved in various disease states. Clin Chim Acta. 2015;446:105-10.
184. Morgan BP. Complement regulatory molecules: application to therapy and transplantation. Immunol Today. 1995;16(6):257-9.
185. Maio M, Brasoveanu LI, Coral S, Sigalotti L, Lamaj E, Gasparollo A, et al. Structure, distribution, and functional role of protectin (CD59) in complement-susceptibility and in immunotherapy of human malignancies (Review). Int J Oncol. 1998;13(2):305-18.
186. Väkevä A, Lehto T, Takala A, Meri S. Detection of a soluble form of the complement membrane attack complex inhibitor CD59 in plasma after acute myocardial infarction. Scand J Immunol. 2000;52(4):411-4.
187. Lehto T, Honkanen E, Teppo AM, Meri S. Urinary excretion of protectin (CD59), complement SC5b-9 and cytokines in membranous glomerulonephritis. Kidney Int. 1995;47(5):1403-11.
188. Meri S, Lehto T, Sutton CW, Tyynelä J, Baumann M. Structural composition and functional characterization of soluble CD59: heterogeneity of the oligosaccharide and glycophosphoinositol (GPI) anchor revealed by laser-desorption mass spectrometric analysis. Biochem J. 1996;316 (Pt 3):923-35.
189. Gehrs KM, Jackson JR, Brown EN, Allikmets R, Hageman GS. Complement, age-related macular degeneration and a vision of the future. Arch Ophthalmol. 2010;128(3):349-58.

190. Gerl VB, Bohl J, Pitz S, Stoffelns B, Pfeiffer N, Bhakdi S. Extensive deposits of complement C3d and C5b-9 in the choriocapillaris of eyes of patients with diabetic retinopathy. Invest Ophthalmol Vis Sci. 2002;43(4):1104-8.
191. Nevo Y, Ben-Zeev B, Tabib A, Straussberg R, Anikster Y, Shorer Z, et al. CD59 deficiency is associated with chronic hemolysis and childhood relapsing immune-mediated polyneuropathy. Blood. 2013;121(1):129-35.
192. Rosoklija GB, Dwork AJ, Younger DS, Karlikaya G, Latov N, Hays AP. Local activation of the complement system in endoneurial microvessels of diabetic neuropathy. Acta Neuropathol. 2000;99(1):55-62.
193. Falk RJ, Scheinman JI, Mauer SM, Michael AF. Polyantigenic expansion of basement membrane constituents in diabetic nephropathy. Diabetes. 1983;32 Suppl 2:34-9.
194. Qin X, Goldfine A, Krumrei N, Grubissich L, Acosta J, Chorev M, et al. Glycation inactivation of the complement regulatory protein CD59: a possible role in the pathogenesis of the vascular complications of human diabetes. Diabetes. 2004;53(10):2653-61.
195. Acosta J, Hettinga J, Flückiger R, Krumrei N, Goldfine A, Angarita L, et al. Molecular basis for a link between complement and the vascular complications of diabetes. Proc Natl Acad Sci U S A. 2000;97(10):5450-5.
196. Ghosh P, Sahoo R, Vaidya A, Cantel S, Kavishwar A, Goldfine A, et al. A specific and sensitive assay for blood levels of glycated CD59: a novel biomarker for diabetes. Am J Hematol. 2013;88(8):670-6.
197. Ghosh P, Vaidya A, Sahoo R, Goldfine A, Herring N, Bry L, et al. Glycation of the complement regulatory protein CD59 is a novel biomarker for glucose handling in humans. J Clin Endocrinol Metab. 2014;99(6):E999-E1006.
198. Ma D, Luque-Fernandez MA, Bogdanet D, Desoye G, Dunne F, Halperin JA. Plasma Glycated CD59 Predicts Early Gestational Diabetes and Large for Gestational Age Newborns. J Clin Endocrinol Metab. 2020;105(4).
199. Ghosh P, Luque-Fernandez MA, Vaidya A, Ma D, Sahoo R, Chorev M, et al. Plasma Glycated CD59, a Novel Biomarker for Detection of Pregnancy-Induced Glucose Intolerance. Diabetes Care. 2017;40(7):981-4.
200. Bogdanet D, O'Shea PM, Halperin J, Dunne F. Plasma glycated CD59 (gCD59), a novel biomarker for the diagnosis, management and follow up of women with Gestational Diabetes (GDM) - protocol for prospective cohort study. BMC Pregnancy Childbirth. 2020;20(1):412.
201. Licht P, Lösch A, Dittrich R, Neuwinger J, Siebzehnrübl E, Wildt L. Novel insights into human endometrial paracrinology and embryo-maternal communication by intrauterine microdialysis. Hum Reprod Update. 1998;4(5):532-8.
202. Ma Q, Fan J, Wang J, Yang S, Cong Q, Wang R, et al. High levels of chorionic gonadotrophin attenuate insulin sensitivity and promote inflammation in adipocytes. J Mol Endocrinol. 2015;54(2):161-70.
203. Sirikunalai P, Wanapirak C, Sirichotiyakul S, Tongprasert F, Srisupundit K, Luewan S, et al. Associations between maternal serum free beta human chorionic gonadotropin (β-hCG) levels and adverse pregnancy outcomes. J Obstet Gynaecol. 2016;36(2):178-82.
204. Ong CYT, Liao AW, Spencer K, Munim S, Nicolaides KH. First trimester maternal serum free β human chorionic gonadotrophin and pregnancy associated plasma protein A as predictors of pregnancy complications. BJOG: An International Journal of Obstetrics & Gynaecology. 2000;107(10):1265-70.
205. WHO Expert Committee on Diabetes Mellitus: second report. World Health Organ Tech Rep Ser. 1980;646:1-80.
206. Xiong F, Li G, Sun Q, Chen P, Wang Z, Wan C, et al. Obstetric and perinatal outcomes of pregnancies according to initial maternal serum HCG concentrations after vitrified-warmed single blastocyst transfer. Reprod Biomed Online. 2019;38(3):455-64.

207. Yue CY, Zhang CY, Ying CM. Serum markers in quadruple screening associated with adverse pregnancy outcomes: A case-control study in China. Clin Chim Acta. 2020;511:278-81.
208. Tul N, Pusenjak S, Osredkar J, Spencer K, Novak-Antolic Z. Predicting complications of pregnancy with first-trimester maternal serum free-betahCG, PAPP-A and inhibin-A. Prenat Diagn. 2003;23(12):990-6.
209. Savvidou MD, Syngelaki A, Muhaisen M, Emelyanenko E, Nicolaides KH. First trimester maternal serum free β-human chorionic gonadotropin and pregnancy-associated plasma protein A in pregnancies complicated by diabetes mellitus. BJOG. 2012;119(4):410-6.
210. Beneventi F, Simonetta M, Lovati E, Albonico G, Tinelli C, Locatelli E, et al. First trimester pregnancy-associated plasma protein-A in pregnancies complicated by subsequent gestational diabetes. Prenat Diagn. 2011;31(6):523-8.
211. Sweeting AN, Wong J, Appelblom H, Ross GP, Kouru H, Williams PF, et al. A first trimester prediction model for gestational diabetes utilizing aneuploidy and pre-eclampsia screening markers. J Matern Fetal Neonatal Med. 2018;31(16):2122-30.
212. Hammond GL, Bocchinfuso WP. Sex hormone-binding globulin: gene organization and structure/function analyses. Horm Res. 1996;45(3-5):197-201.
213. Hammond GL. Diverse roles for sex hormone-binding globulin in reproduction. Biol Reprod. 2011;85(3):431-41.
214. Glass AR, Swerdloff RS, Bray GA, Dahms WT, Atkinson RL. Low serum testosterone and sex-hormone-binding-globulin in massively obese men. J Clin Endocrinol Metab. 1977;45(6):1211-9.
215. Guzick DS, Wing R, Smith D, Berga SL, Winters SJ. Endocrine consequences of weight loss in obese, hyperandrogenic, anovulatory women. Fertil Steril. 1994;61(4):598-604.
216. Hammoud A, Gibson M, Hunt SC, Adams TD, Carrell DT, Kolotkin RL, et al. Effect of Roux-en-Y gastric bypass surgery on the sex steroids and quality of life in obese men. J Clin Endocrinol Metab. 2009;94(4):1329-32.
217. Pitteloud N, Mootha VK, Dwyer AA, Hardin M, Lee H, Eriksson KF, et al. Relationship between testosterone levels, insulin sensitivity, and mitochondrial function in men. Diabetes Care. 2005;28(7):1636-42.
218. Kajaia N, Binder H, Dittrich R, Oppelt PG, Flor B, Cupisti S, et al. Low sex hormone-binding globulin as a predictive marker for insulin resistance in women with hyperandrogenic syndrome. Eur J Endocrinol. 2007;157(4):499-507.
219. Laaksonen DE, Niskanen L, Punnonen K, Nyyssönen K, Tuomainen TP, Salonen R, et al. Sex hormones, inflammation and the metabolic syndrome: a population-based study. Eur J Endocrinol. 2003;149(6):601-8.
220. Brand JS, van der Tweel I, Grobbee DE, Emmelot-Vonk MH, van der Schouw YT. Testosterone, sex hormone-binding globulin and the metabolic syndrome: a systematic review and meta-analysis of observational studies. Int J Epidemiol. 2011;40(1):189-207.
221. Jaruvongvanich V, Sanguankeo A, Riangwiwat T, Upala S. Testosterone, Sex Hormone-Binding Globulin and Nonalcoholic Fatty Liver Disease: a Systematic Review and Meta-Analysis. Ann Hepatol. 2017;16(3):382-94.
222. Ding EL, Song Y, Malik VS, Liu S. Sex differences of endogenous sex hormones and risk of type 2 diabetes: a systematic review and meta-analysis. JAMA. 2006;295(11):1288-99.
223. Hu J, Zhang A, Yang S, Wang Y, Goswami R, Zhou H, et al. Combined effects of sex hormone-binding globulin and sex hormones on risk of incident type 2 diabetes. J Diabetes. 2016;8(4):508-15.
224. Muka T, Nano J, Jaspers L, Meun C, Bramer WM, Hofman A, et al. Associations of Steroid Sex Hormones and Sex Hormone-Binding Globulin With the Risk of Type 2 Diabetes in Women: A Population-Based Cohort Study and Meta-analysis. Diabetes. 2017;66(3):577-86.
225. Pugeat M, Crave JC, Elmidani M, Nicolas MH, Garoscio-Cholet M, Lejeune H, et al. Pathophysiology of sex hormone binding globulin (SHBG): relation to insulin. J Steroid Biochem Mol Biol. 1991;40(4-6):841-9.

226. Plymate SR, Matej LA, Jones RE, Friedl KE. Inhibition of sex hormone-binding globulin production in the human hepatoma (Hep G2) cell line by insulin and prolactin. J Clin Endocrinol Metab. 1988;67(3):460-4.
227. Winters SJ, Gogineni J, Karegar M, Scoggins C, Wunderlich CA, Baumgartner R, et al. Sex hormone-binding globulin gene expression and insulin resistance. J Clin Endocrinol Metab. 2014;99(12):E2780-8.
228. Shin JY, Kim SK, Lee MY, Kim HS, Ye BI, Shin YG, et al. Serum sex hormone-binding globulin levels are independently associated with nonalcoholic fatty liver disease in people with type 2 diabetes. Diabetes Res Clin Pract. 2011;94(1):156-62.
229. Flechtner-Mors M, Schick A, Oeztuerk S, Haenle MM, Wilhelm M, Koenig W, et al. Associations of fatty liver disease and other factors affecting serum SHBG concentrations: a population based study on 1657 subjects. Horm Metab Res. 2014;46(4):287-93.
230. Veltman-Verhulst SM, van Haeften TW, Eijkemans MJ, de Valk HW, Fauser BC, Goverde AJ. Sex hormone-binding globulin concentrations before conception as a predictor for gestational diabetes in women with polycystic ovary syndrome. Hum Reprod. 2010;25(12):3123-8.
231. Hedderson MM, Xu F, Darbinian JA, Quesenberry CP, Sridhar S, Kim C, et al. Prepregnancy SHBG concentrations and risk for subsequently developing gestational diabetes mellitus. Diabetes Care. 2014;37(5):1296-303.
232. Badon SE, Zhu Y, Sridhar SB, Xu F, Lee C, Ehrlich SF, et al. A Pre-Pregnancy Biomarker Risk Score Improves Prediction of Future Gestational Diabetes. J Endocr Soc. 2018;2(10):1158-69.
233. Li MY, Rawal S, Hinkle SN, Zhu YY, Tekola-Ayele F, Tsai MY, et al. Sex Hormone-binding Globulin, Cardiometabolic Biomarkers, and Gestational Diabetes: A Longitudinal Study and Meta-analysis. Matern Fetal Med. 2020;2(1):2-9.
234. Smirnakis KV, Plati A, Wolf M, Thadhani R, Ecker JL. Predicting gestational diabetes: choosing the optimal early serum marker. Am J Obstet Gynecol. 2007;196(4):410.e1-6; discussion .e6-7.
235. Bulletins--Obstetrics ACoOaGCoP. ACOG Practice Bulletin. Clinical management guidelines for obstetrician-gynecologists. Number 30, September 2001 (replaces Technical Bulletin Number 200, December 1994). Gestational diabetes. Obstet Gynecol. 2001;98(3):525-38.
236. Caglar GS, Ozdemir ED, Cengiz SD, Demirtaş S. Sex-hormone-binding globulin early in pregnancy for the prediction of severe gestational diabetes mellitus and related complications. J Obstet Gynaecol Res. 2012;38(11):1286-93.
237. Maged AM, Moety GA, Mostafa WA, Hamed DA. Comparative study between different biomarkers for early prediction of gestational diabetes mellitus. J Matern Fetal Neonatal Med. 2014;27(11):1108-12.
238. Tawfeek MA, Alfadhli EM, Alayoubi AM, El-Beshbishy HA, Habib FA. Sex hormone binding globulin as a valuable biochemical marker in predicting gestational diabetes mellitus. BMC Womens Health. 2017;17(1):18.
239. Siddiqui K, George TP, Joy SS, Nawaz SS. Association of sex hormone binding globulin with gestational age and parity in gestational diabetes mellitus. J Matern Fetal Neonatal Med. 2020:1-6.
240. Ajjan R, Carter AM, Somani R, Kain K, Grant PJ. Ethnic differences in cardiovascular risk factors in healthy Caucasian and South Asian individuals with the metabolic syndrome. J Thromb Haemost. 2007;5(4):754-60.
241. McElduff A, Hitchman R, McElduff P. Is sex hormone-binding globulin associated with glucose tolerance? Diabet Med. 2006;23(3):306-12.
242. Key TJ, Pike MC, Moore JW, Wang DY, Morgan B. The relationship of free fatty acids with the binding of oestradiol to SHBG and to albumin in women. J Steroid Biochem. 1990;35(1):35-8.
243. Hamilton-Fairley D, White D, Griffiths M, Anyaoku V, Koistinen R, Seppälä M, et al. Diurnal variation of sex hormone binding globulin and insulin-like growth factor binding protein-1 in women with polycystic ovary syndrome. Clin Endocrinol (Oxf). 1995;43(2):159-65.
244. Catalano PM. Carbohydrate metabolism and gestational diabetes. Clin Obstet Gynecol. 1994;37(1):25-38.

245. Stefan N, Kantartzis K, Häring HU. Causes and metabolic consequences of Fatty liver. Endocr Rev. 2008;29(7):939-60.
246. Venugopal SK, Devaraj S, Jialal I. Macrophage conditioned medium induces the expression of C-reactive protein in human aortic endothelial cells: potential for paracrine/autocrine effects. Am J Pathol. 2005;166(4):1265-71.
247. Ganter U, Arcone R, Toniatti C, Morrone G, Ciliberto G. Dual control of C-reactive protein gene expression by interleukin-1 and interleukin-6. EMBO J. 1989;8(12):3773-9.
248. Sproston NR, Ashworth JJ. Role of C-Reactive Protein at Sites of Inflammation and Infection. Front Immunol. 2018;9:754.
249. Retnakaran R, Hanley AJ, Raif N, Connelly PW, Sermer M, Zinman B. C-reactive protein and gestational diabetes: the central role of maternal obesity. J Clin Endocrinol Metab. 2003;88(8):3507-12.
250. Jabs WJ, Lögering BA, Gerke P, Kreft B, Wolber EM, Klinger MH, et al. The kidney as a second site of human C-reactive protein formation in vivo. Eur J Immunol. 2003;33(1):152-61.
251. Kim SH, Reaven G, Lindley S. Relationship between insulin resistance and C-reactive protein in a patient population treated with second generation antipsychotic medications. Int Clin Psychopharmacol. 2011;26(1):43-7.
252. Moran A, Steffen LM, Jacobs DR, Steinberger J, Pankow JS, Hong CP, et al. Relation of C-reactive protein to insulin resistance and cardiovascular risk factors in youth. Diabetes Care. 2005;28(7):1763-8.
253. Yan Y, Li S, Liu Y, Bazzano L, He J, Mi J, et al. Temporal relationship between inflammation and insulin resistance and their joint effect on hyperglycemia: the Bogalusa Heart Study. Cardiovasc Diabetol. 2019;18(1):109.
254. Ridker PM, Buring JE, Cook NR, Rifai N. C-reactive protein, the metabolic syndrome, and risk of incident cardiovascular events: an 8-year follow-up of 14 719 initially healthy American women. Circulation. 2003;107(3):391-7.
255. Aronson D, Bartha P, Zinder O, Kerner A, Markiewicz W, Avizohar O, et al. Obesity is the major determinant of elevated C-reactive protein in subjects with the metabolic syndrome. Int J Obes Relat Metab Disord. 2004;28(5):674-9.
256. Visser M, Bouter LM, McQuillan GM, Wener MH, Harris TB. Elevated C-reactive protein levels in overweight and obese adults. JAMA. 1999;282(22):2131-5.
257. Kahn SE, Zinman B, Haffner SM, O'Neill MC, Kravitz BG, Yu D, et al. Obesity is a major determinant of the association of C-reactive protein levels and the metabolic syndrome in type 2 diabetes. Diabetes. 2006;55(8):2357-64.
258. Alamolhoda SH, Yazdkhasti M, Namdari M, Zakariayi SJ, Mirabi P. Association between C-reactive protein and gestational diabetes: a prospective study. J Obstet Gynaecol. 2020;40(3):349-53.
259. Savvidou M, Nelson SM, Makgoba M, Messow CM, Sattar N, Nicolaides K. First-trimester prediction of gestational diabetes mellitus: examining the potential of combining maternal characteristics and laboratory measures. Diabetes. 2010;59(12):3017-22.
260. Wolf M, Sandler L, Hsu K, Vossen-Smirnakis K, Ecker JL, Thadhani R. First-trimester C-reactive protein and subsequent gestational diabetes. Diabetes Care. 2003;26(3):819-24.
261. Alyas S, Roohi N, Ashraf S, Ilyas S, Ilyas A. Early pregnancy biochemical markers of placentation for screening of gestational diabetes mellitus (GDM). Diabetes Metab Syndr. 2019;13(4):2353-6.
262. Korkmazer E, Solak N. Correlation between inflammatory markers and insulin resistance in pregnancy. J Obstet Gynaecol. 2015;35(2):142-5.
263. Corcoran SM, Achamallah N, Loughlin JO, Stafford P, Dicker P, Malone FD, et al. First trimester serum biomarkers to predict gestational diabetes in a high-risk cohort: Striving for clinically useful thresholds. Eur J Obstet Gynecol Reprod Biol. 2018;222:7-12.

264. Adam S, Pheiffer C, Dias S, Rheeder P. Association between gestational diabetes and biomarkers: a role in diagnosis. Biomarkers. 2018;23(4):386-91.
265. Classification and diagnosis of diabetes mellitus and other categories of glucose intolerance. National Diabetes Data Group. Diabetes. 1979;28(12):1039-57.
266. Amirian A, Rahnemaei FA, Abdi F. Role of C-reactive Protein(CRP) or high-sensitivity CRP in predicting gestational diabetes Mellitus:Systematic review. Diabetes Metab Syndr. 2020;14(3):229-36.
267. Oh-I S, Shimizu H, Satoh T, Okada S, Adachi S, Inoue K, et al. Identification of nesfatin-1 as a satiety molecule in the hypothalamus. Nature. 2006;443(7112):709-12.
268. Su Y, Zhang J, Tang Y, Bi F, Liu JN. The novel function of nesfatin-1: anti-hyperglycemia. Biochem Biophys Res Commun. 2010;391(1):1039-42.
269. Dong J, Xu H, Wang PF, Cai GJ, Song HF, Wang CC, et al. Nesfatin-1 stimulates fatty-acid oxidation by activating AMP-activated protein kinase in STZ-induced type 2 diabetic mice. PLoS One. 2013;8(12):e83397.
270. Li QC, Wang HY, Chen X, Guan HZ, Jiang ZY. Fasting plasma levels of nesfatin-1 in patients with type 1 and type 2 diabetes mellitus and the nutrient-related fluctuation of nesfatin-1 level in normal humans. Regul Pept. 2010;159(1-3):72-7.
271. Zhai T, Li SZ, Fan XT, Tian Z, Lu XQ, Dong J. Circulating Nesfatin-1 Levels and Type 2 Diabetes: A Systematic Review and Meta-Analysis. J Diabetes Res. 2017;2017:7687098.
272. Kucukler FK, Gorkem U, Simsek Y, Kocabas R, Gulen S, Guler S. Low level of Nesfatin-1 is associated with gestational diabetes mellitus. Gynecol Endocrinol. 2016;32(9):759-61.
273. Ademoglu EN, Gorar S, Keskin M, Carlioglu A, Ucler R, Erdamar H, et al. Serum nesfatin-1 levels are decreased in pregnant women newly diagnosed with gestational diabetes. Arch Endocrinol Metab. 2017;61(5):455-9.
274. Mierzyński R, Poniedziałek-Czajkowska E, Dłuski D, Patro-Małysza J, Kimber-Trojnar Ż, Majsterek M, et al. Nesfatin-1 and Vaspin as Potential Novel Biomarkers for the Prediction and Early Diagnosis of Gestational Diabetes Mellitus. Int J Mol Sci. 2019;20(1).
275. Fialova L, Malbohan IM. Pregnancy-associated plasma protein A (PAPP-A): theoretical and clinical aspects. Bratisl Lek Listy. 2002;103(6):194-205.
276. Dugoff L, Hobbins JC, Malone FD, Porter TF, Luthy D, Comstock CH, et al. First-trimester maternal serum PAPP-A and free-beta subunit human chorionic gonadotropin concentrations and nuchal translucency are associated with obstetric complications: a population-based screening study (the FASTER Trial). Am J Obstet Gynecol. 2004;191(4):1446-51.
277. Leguy MC, Brun S, Pidoux G, Salhi H, Choiset A, Menet MC, et al. Pattern of secretion of pregnancy-associated plasma protein-A (PAPP-A) during pregnancies complicated by fetal aneuploidy, in vivo and in vitro. Reprod Biol Endocrinol. 2014;12:129.
278. Pellitero S, Reverter JL, Pizarro E, Pastor MC, Granada ML, Tàssies D, et al. Pregnancy-associated plasma protein-a levels are related to glycemic control but not to lipid profile or hemostatic parameters in type 2 diabetes. Diabetes Care. 2007;30(12):3083-5.
279. Resch ZT, Chen BK, Bale LK, Oxvig C, Overgaard MT, Conover CA. Pregnancy-associated plasma protein a gene expression as a target of inflammatory cytokines. Endocrinology. 2004;145(3):1124-9.
280. Lovati E, Beneventi F, Simonetta M, Laneri M, Quarleri L, Scudeller L, et al. Gestational diabetes mellitus: including serum pregnancy-associated plasma protein-A testing in the clinical management of primiparous women? A case-control study. Diabetes Res Clin Pract. 2013;100(3):340-7.
281. Ramezani S, Doulabi MA, Saqhafi H, Alipoor M. Prediction of Gestational Diabetes by Measuring the Levels of Pregnancy Associated Plasma Protein-A (PAPP-A) During Gestation Weeks 11-14. J Reprod Infertil. 2020;21(2):130-7.

282. Ren Z, Zhe D, Li Z, Sun XP, Yang K, Lin L. Study on the correlation and predictive value of serum pregnancy-associated plasma protein A, triglyceride and serum 25-hydroxyvitamin D levels with gestational diabetes mellitus. World J Clin Cases. 2020;8(5):864-73.
283. Snyder BM, Baer RJ, Oltman SP, Robinson JG, Breheny PJ, Saftlas AF, et al. Early pregnancy prediction of gestational diabetes mellitus risk using prenatal screening biomarkers in nulliparous women. Diabetes Res Clin Pract. 2020;163:108139.
284. Xiao D, Chenhong W, Yanbin X, Lu Z. Gestational diabetes mellitus and first trimester pregnancy-associated plasma protein A: A case-control study in a Chinese population. J Diabetes Investig. 2018;9(1):204-10.
285. Syngelaki A, Kotecha R, Pastides A, Wright A, Nicolaides KH. First-trimester biochemical markers of placentation in screening for gestational diabetes mellitus. Metabolism. 2015;64(11):1485-9.
286. Donovan BM, Nidey NL, Jasper EA, Robinson JG, Bao W, Saftlas AF, et al. First trimester prenatal screening biomarkers and gestational diabetes mellitus: A systematic review and meta-analysis. PLoS One. 2018;13(7):e0201319.
287. Wells G, Bleicher K, Han X, McShane M, Chan YF, Bartlett A, et al. Maternal Diabetes, Large-for-Gestational-Age Births, and First Trimester Pregnancy-Associated Plasma Protein-A. J Clin Endocrinol Metab. 2015;100(6):2372-9.
288. Jayabalan N, Lai A, Nair S, Guanzon D, Scholz-Romero K, Palma C, et al. Quantitative Proteomics by SWATH-MS Suggest an Association Between Circulating Exosomes and Maternal Metabolic Changes in Gestational Diabetes Mellitus. Proteomics. 2019;19(1-2):e1800164.
289. Alapatt P, Guo F, Komanetsky SM, Wang S, Cai J, Sargsyan A, et al. Liver retinol transporter and receptor for serum retinol-binding protein (RBP4). J Biol Chem. 2013;288(2):1250-65.
290. Majerczyk M, Olszanecka-Glinianowicz M, Puzianowska-Kuźnicka M, Chudek J. Retinol-binding protein 4 (RBP4) as the causative factor and marker of vascular injury related to insulin resistance. Postepy Hig Med Dosw (Online). 2016;70(0):1267-75.
291. Yang Q, Graham TE, Mody N, Preitner F, Peroni OD, Zabolotny JM, et al. Serum retinol binding protein 4 contributes to insulin resistance in obesity and type 2 diabetes. Nature. 2005;436(7049):356-62.
292. Jin C, Lin L, Han N, Zhao Z, Liu Z, Luo S, et al. Plasma retinol-binding protein 4 in the first and second trimester and risk of gestational diabetes mellitus in Chinese women: a nested case-control study. Nutr Metab (Lond). 2020;17:1.
293. Yuan XS, Shi H, Wang HY, Yu B, Jiang J. Ficolin-3/adiponectin ratio for the prediction of gestational diabetes mellitus in pregnant women. J Diabetes Investig. 2018;9(2):403-10.
294. Du X, Dong Y, Xiao L, Liu GH, Qin W, Yu H. Association between retinol-binding protein 4 concentrations and gestational diabetes mellitus (A1GDM and A2GDM) in different pregnancy and postpartum periods. Ann Transl Med. 2019;7(18):479.
295. Khovidhunkit W, Pruksakorn P, Plengpanich W, Tharavanij T. Retinol-binding protein 4 is not associated with insulin resistance in pregnancy. Metabolism. 2012;61(1):65-9.
296. Huang QT, Huang Q, Luo W, Li F, Hang LL, Yu YH, et al. Circulating retinol-binding protein 4 levels in gestational diabetes mellitus: a meta-analysis of observational studies. Gynecol Endocrinol. 2015;31(5):337-44.
297. Hu S, Liu Q, Huang X, Tan H. Serum level and polymorphisms of retinol-binding protein-4 and risk for gestational diabetes mellitus: a meta-analysis. BMC Pregnancy Childbirth. 2016;16:52.
298. Jia J, Bai J, Liu Y, Yin J, Yang P, Yu S, et al. Association between retinol-binding protein 4 and polycystic ovary syndrome: a meta-analysis. Endocr J. 2014;61(10):995-1002.
299. Kim SH, Choi HJ, Im JA. Retinol-binding protein 4 responses during an oral glucose tolerance testing in women with gestational diabetes mellitus. Clin Chim Acta. 2008;391(1-2):123-5.
300. Chan TF, Chen HS, Chen YC, Lee CH, Chou FH, Chen IJ, et al. Increased serum retinol-binding protein 4 concentrations in women with gestational diabetes mellitus. Reprod Sci. 2007;14(2):169-74.

301. Zhaoxia L, Mengkai D, Qin F, Danqing C. Significance of RBP4 in patients with gestational diabetes mellitus: a case-control study of Han Chinese women. Gynecol Endocrinol. 2014;30(2):161-4.
302. Su YX, Hong J, Yan Q, Xu C, Gu WQ, Zhang YF, et al. Increased serum retinol-binding protein-4 levels in pregnant women with and without gestational diabetes mellitus. Diabetes Metab. 2010;36(6 Pt 1):470-5.
303. Tepper BJ, Kim YK, Shete V, Shabrova E, Quadro L. Serum retinol-binding protein 4 (RBP4) and retinol in a cohort of borderline obese women with and without gestational diabetes. Clin Biochem. 2010;43(3):320-3.
304. Krzyzanowska K, Zemany L, Krugluger W, Schernthaner GH, Mittermayer F, Schnack C, et al. Serum concentrations of retinol-binding protein 4 in women with and without gestational diabetes. Diabetologia. 2008;51(7):1115-22.
305. Lewandowski KC, Stojanovic N, Bienkiewicz M, Tan BK, Prelevic GM, Press M, et al. Elevated concentrations of retinol-binding protein-4 (RBP-4) in gestational diabetes mellitus: negative correlation with soluble vascular cell adhesion molecule-1 (sVCAM-1). Gynecol Endocrinol. 2008;24(6):300-5.
306. Abetew DF, Qiu C, Fida NG, Dishi M, Hevner K, Williams MA, et al. Association of retinol binding protein 4 with risk of gestational diabetes. Diabetes Res Clin Pract. 2013;99(1):48-53.
307. Liu M, Chen Y, Chen D. Association between transthyretin concentrations and gestational diabetes mellitus in Chinese women. Arch Gynecol Obstet. 2020;302(2):329-35.
308. Graham TE, Wason CJ, Blüher M, Kahn BB. Shortcomings in methodology complicate measurements of serum retinol binding protein (RBP4) in insulin-resistant human subjects. Diabetologia. 2007;50(4):814-23.
309. Idris N, Hatikah CC, Murizah M, Rushdan M. Universal versus selective screening for detection of gestational diabetes mellitus in a malaysian population. Malays Fam Physician. 2009;4(2-3):83-7.
310. Avalos GE, Owens LA, Dunne F, Collaborators AD. Applying current screening tools for gestational diabetes mellitus to a European population: is it time for change? Diabetes Care. 2013;36(10):3040-4.
311. Alberico S, Strazzanti C, De Santo D, De Seta F, Lenardon P, Bernardon M, et al. Gestational diabetes: universal or selective screening? J Matern Fetal Neonatal Med. 2004;16(6):331-7.
312. Kuo CH, Li HY. Diagnostic Strategies for Gestational Diabetes Mellitus: Review of Current Evidence. Curr Diab Rep. 2019;19(12):155.
313. Mo X, Gai Tobe R, Takahashi Y, Arata N, Liabsuetrakul T, Nakayama T, et al. Economic Evaluations of Gestational Diabetes Mellitus Screening: A Systematic Review. J Epidemiol. 2021;31(3):220-30.
314. Danyliv A, Gillespie P, O'Neill C, Tierney M, O'Dea A, McGuire BE, et al. The cost-effectiveness of screening for gestational diabetes mellitus in primary and secondary care in the Republic of Ireland. Diabetologia. 2016;59(3):436-44.
315. Di Cianni G, Volpe L, Casadidio I, Bottone P, Marselli L, Lencioni C, et al. Universal screening and intensive metabolic management of gestational diabetes: cost-effectiveness in Italy. Acta Diabetol. 2002;39(2):69-73.
316. Miailhe G, Kayem G, Girard G, Legardeur H, Mandelbrot L. Selective rather than universal screening for gestational diabetes mellitus? Eur J Obstet Gynecol Reprod Biol. 2015;191:95-100.
317. Cosson E, Benbara A, Pharisien I, Nguyen MT, Revaux A, Lormeau B, et al. Diagnostic and prognostic performances over 9 years of a selective screening strategy for gestational diabetes mellitus in a cohort of 18,775 subjects. Diabetes Care. 2013;36(3):598-603.
318. Wen SW, Liu S, Kramer MS, Joseph KS, Levitt C, Marcoux S, et al. Impact of prenatal glucose screening on the diagnosis of gestational diabetes and on pregnancy outcomes. Am J Epidemiol. 2000;152(11):1009-14; discussion 15-6.

319. Cosson E, Benchimol M, Carbillon L, Pharisien I, Pariès J, Valensi P, et al. Universal rather than selective screening for gestational diabetes mellitus may improve fetal outcomes. Diabetes Metab. 2006;32(2):140-6.
320. Farrar D, Fairley L, Wright J, Tuffnell D, Whitelaw D, Lawlor DA. Evaluation of the impact of universal testing for gestational diabetes mellitus on maternal and neonatal health outcomes: a retrospective analysis. BMC Pregnancy Childbirth. 2014;14:317.
321. Griffin ME, Coffey M, Johnson H, Scanlon P, Foley M, Stronge J, et al. Universal vs. risk factor-based screening for gestational diabetes mellitus: detection rates, gestation at diagnosis and outcome. Diabet Med. 2000;17(1):26-32.
322. Sweeting AN, Ross GP, Hyett J, Molyneaux L, Constantino M, Harding AJ, et al. Gestational Diabetes Mellitus in Early Pregnancy: Evidence for Poor Pregnancy Outcomes Despite Treatment. Diabetes Care. 2016;39(1):75-81.
323. Most OL, Kim JH, Arslan AA, Klauser C. Maternal and neonatal outcomes in early glucose tolerance testing in an obstetric population in New York city. J Perinat Med. 2009;37(2):114-7.
324. Simmons D, Nema J, Parton C, Vizza L, Robertson A, Rajagopal R, et al. The treatment of booking gestational diabetes mellitus (TOBOGM) pilot randomised controlled trial. BMC Pregnancy Childbirth. 2018;18(1):151.
325. Zhu Z, Cao F, Li X. Epigenetic Programming and Fetal Metabolic Programming. Front Endocrinol (Lausanne). 2019;10:764.
326. Mayeux R. Biomarkers: potential uses and limitations. NeuroRx. 2004;1(2):182-8.
327. Mahdi T, Hänzelmann S, Salehi A, Muhammed SJ, Reinbothe TM, Tang Y, et al. Secreted frizzled-related protein 4 reduces insulin secretion and is overexpressed in type 2 diabetes. Cell Metab. 2012;16(5):625-33.
328. Schuitemaker JHN, Beernink RHJ, Franx A, Cremers TIFH, Koster MPH. First trimester secreted Frizzled-Related Protein 4 and other adipokine serum concentrations in women developing gestational diabetes mellitus. PLoS One. 2020;15(11):e0242423.
329. Baldane S, Ipekci SH, Kebapcilar AG, Abusoglu A, Beyhekim H, Ilhan TT, et al. Prorenin and secreted frizzled-related protein 4 levels in women with gestational diabetes mellitus. Bratisl Lek Listy. 2018;119(7):450-3.
330. Yuan XS, Zhang M, Wang HY, Jiang J, Yu B. Increased secreted frizzled-related protein 4 and ficolin-3 levels in gestational diabetes mellitus women. Endocr J. 2018;65(4):499-508.
331. Amini M, Kazemnejad A, Zayeri F, Montazeri A, Rasekhi A, Amirian A, et al. Diagnostic accuracy of maternal serum multiple marker screening for early detection of gestational diabetes mellitus in the absence of a gold standard test. BMC Pregnancy Childbirth. 2020;20(1):375.
332. Liu L, Hu J, Yang L, Wang N, Liu Y, Wei X, et al. Association of WISP1/CCN4 with Risk of Overweight and Gestational Diabetes Mellitus in Chinese Pregnant Women. Dis Markers. 2020;2020:4934206.
333. Sahin Ersoy G, Altun Ensari T, Subas S, Giray B, Simsek EE, Cevik O. WISP1 is a novel adipokine linked to metabolic parameters in gestational diabetes mellitus. J Matern Fetal Neonatal Med. 2017;30(8):942-6.
334. Al-Ghazali MJ, Ali HA, Al-Rufaie MM. Serum irisin levels as a potential marker for diagnosis of gestational diabetes mellitus. Acta Biomed. 2020;91(1):56-63.
335. Onat T, Inandiklioglu N. Circulating Myonectin and Irisin Levels in Gestational Diabetes Mellitus - A Case-control Study. Z Geburtshilfe Neonatol. 2021.
336. Gutaj P, Sibiak R, Jankowski M, Awdi K, Bryl R, Mozdziak P, et al. The Role of the Adipokines in the Most Common Gestational Complications. Int J Mol Sci. 2020;21(24).
337. Zhong L, Long Y, Wang S, Lian R, Deng L, Ye Z, et al. Continuous elevation of plasma asprosin in pregnant women complicated with gestational diabetes mellitus: A nested case-control study. Placenta. 2020;93:17-22.

338. Yavuzkir S, Ugur K, Deniz R, Ustebay DU, Mirzaoglu M, Yardim M, et al. Maternal and umbilical cord blood subfatin and spexin levels in patients with gestational diabetes mellitus. Peptides. 2020;126:170277.
339. Kang L, Li HY, Ou HY, Wu P, Wang SH, Chang CJ, et al. Role of placental fibrinogen-like protein 1 in gestational diabetes. Transl Res. 2020;218:73-80.
340. Liu L, Hu J, Wang N, Liu Y, Wei X, Gao M, et al. A novel association of CCDC80 with gestational diabetes mellitus in pregnant women: a propensity score analysis from a case-control study. BMC Pregnancy Childbirth. 2020;20(1):53.
341. Deischinger C, Leitner K, Baumgartner-Parzer S, Bancher-Todesca D, Kautzky-Willer A, Harreiter J. CTRP-1 levels are related to insulin resistance in pregnancy and gestational diabetes mellitus. Sci Rep. 2020;10(1):17345.
342. The Top 10 research priorities for diabetes in pregnancy Accessed January 7th 2021 [Available from: https://www.jla.nihr.ac.uk/news/the-top-10-research-priorities-for-diabetes-in-pregnancy/26184.

Fig. 1 Prisma diagram

PRISMA 2009 Flow Diagram

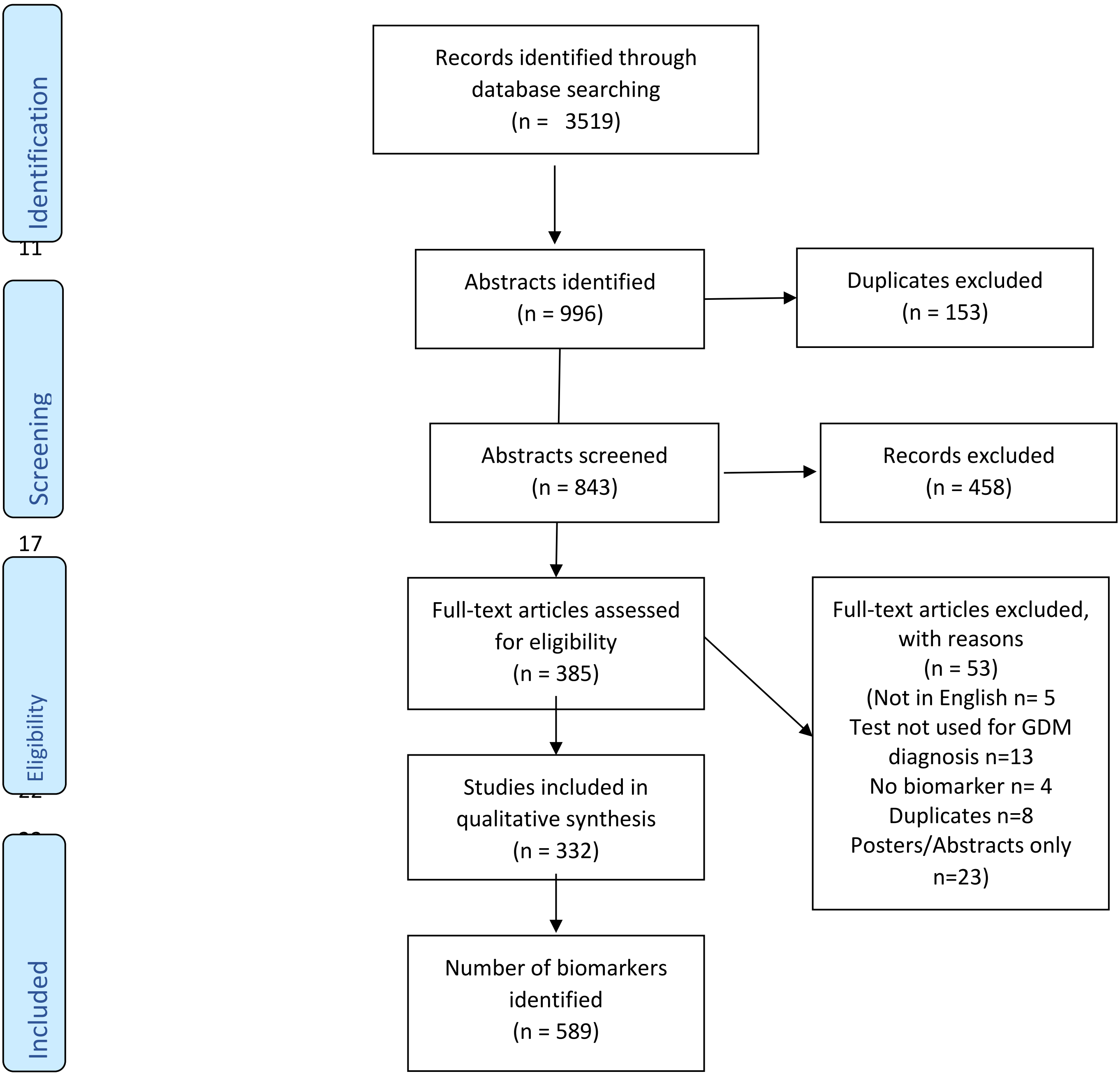

Table 1. Protein biomarkers (n=15) identified post application of post-hoc criteria

Cytokine	Glycoprotein	Protein
*Adiponectin	Afamin	CRP
* Chemerin	CD59	Nefastin-1
* Fetuin	hCG	PAPP-A
* Leptin	SHBG	RBP4
* Omentin		
IL-6		
TNF		

*Adipokines; IL-6: Interleukin 6; TNF: Tumour Necrosis Factor; hCG: Human Chorionic Gonadotrophin; SHBG: Sex Hormone Binding Globulin; CRP: C-Reactive Protein; PAPP-A: Placental Associated Plasma Protein A; RBP4: Retinol Binding Protein 4.

Table 2. Summary of test performance at the time of GDM diagnosis*

Biomarker	First Author (Ref.)	Analytical method	Diagnostic Sensitivity %	Diagnostic Specificity %	AUC	Cut-off value
**Cytokines**						
**Adiponectin**	Bozkurt *et al.* (36)	RIA	NS	ns	0.62	ns
	Weerakiet *et al.* (39)	ELISA	91.7	30.8	0.63	10 µg/mL
**Chemerin**	Wang *et al.* (63)	ELISA	73.3	76	0.82	6.78 µg/L
**Leptin**	Bozkurt *et al.* (36)	RIA	ns	ns	0.61	ns
	Boyadzhieva *et al.* (103)	ELISA	81.2	64.2	0.82	28.7 ng/mL
**Glycoproteins**						
**CD59**	Gosh *et al.* (198)	ELISA	85	92	0.92	ns
	Ma *et al.* (199)	ELISA	54	93	0.86	ns
**SHBG**	Tawfeek *et al.* (230)	ELISA	96	95	0.91	50 nmol/L
**Other Proteins**						
**RBP4**	Du *et al.* (294)	ELISA	79.4	79.1	0.87	34.84 µg/mL)

* no information on test performance at the time of GDM diagnosis was found for the following biomarkers: fetuin, omentin, IL-6, TNF, afamin, hCG, CRP, Nesfatin-1, PAPP-A.

AUC: area under the curve; IL-6: Interleukin 6; TNF: Tumor Necrosis Factor; hCG: Human Chorionic Gonadotropin; SHBG: Sex Hormone Binding Protein; CRP: C-Reactive Protein; PAPP-A: Pregnancy-Associated Plasma Protein A; RBP4: Retinol Binding Protein 4; RIA: Radioimmunoassay; ELISA: Enzyme-Linked Immunosorbent Assay; ns: not stated.

Table 3. Summary of test performance as a predictive indicator of GDM*

Biomarker	First Author, year (Ref.)	Analytical method	Diagnostic Sensitivity %	Diagnostic Specificity %	AUC	Cut-off value
**Cytokines**						
**Adiponectin**	Georgiou *et al.* (42)	ELISA	85[1]	85.7[1]	0.86	3.5 µg/mL
	Ferreira *et al.* (44)	ELISA	ns	ns	0.85[2]	ns
	Madhu *et al.* (45)	ELISA	100	95.6	ns	9.1 µg/mL
	Iliodromiti *et al.* (47) **	ns	64.7	77.8	0.78	ns
**Fetuin**	Kansu-Celik *et al.* (83)	ELISA	58.6	76.2	0.33	166ng/mL
	Jin *et al.* (87)	ELISA	64.4	58.5	0.61	305.9pg/mL
**Leptin**	Bawah *et al.* (104)	ELISA	95.7	68.6	0.81	18.9 ng/mL
**TNF**	Syngelaki *et al.* (163)	ELISA	ns	ns	0.82	ns
**Glycoproteins**						
**Afamin**	Tramontana *et al.* (177)	ELISA	ns	ns	0.66[3]	ns
	Koninger *et al.* (180) **	ELISA	79.3	79.4	0.78	88.6 mg/L
	Ravnsborg *et al.* (181) **	nanoLC-MS	ns	ns	0.67	ns
**SHBG**	Caglar *et al.* (236)	RIA	46.7	84.1	0.87	97.47nmol/L
	Maged *et al.* (237)	ELISA	85.2	37	0.69	211.5nmol/L
	Veltman-Verhulst *et al.* (238) **	ECL	81	82.8	0.86	58.5 nmol/L
	Badon *et al.* (240) **	ELISA	ns	ns	0.71[2]	44.2 nmol/L
**Other Proteins**						
**CRP**	Kansu-Celik *et al.* (83)	Nephelometry	86.2	50.8	0.70	ns
**PAPP-A**	Lovati *et al.* (280)	DELFIA	81.4[2]	50.5[2]	0.70[2]	ns
	Ramezani *et al.* (281)	ELISA	73.3	57.3	0.61	1896 mU/L
	Ramezani *et al.* (281)	ELISA	34.4	83.2	0.62	0.3 mU/L
	Ren *et al.* (282)	TRFIA	72.5	82.3	0.86	16.34 ng/L
	Snyder *et al.* (283)	DELFIA	75.7[2]	55.5[2]	0.71[2]	ns
	Xiao *et al.* (284)	DELFIA	ns	ns	0.53; 0.68[2]	ns
	Syngelaki *et al.* (285)	DELFIA	ns	ns	0.84[2]	ns
**RBP4**	Yuan *et al.*	EIA	63.6	75	0.72	30.45 µg/mL

*no information on test performance as a predictive indicator of GDM was found for the following biomarkers: chemerin, omentin, IL-6, CD59, hCG, Nesfatin-1.

AUC: area under the curve; IL:6: Interleukin 6; TNF: Tumor Necrosis Factor; hCG: Human Chorionic Gonadotropin; SHBG: Sex Hormone Binding Protein; CRP: C-Reactive Protein; PAPP-A: Pregnancy-Associated Plasma Protein A; RBP4: Retinol Binding Protein 4; RIA: Radioimmunoassay; ELISA: Enzyme-Linked Immunosorbent Assay; nanoLC-MS: nano-flow Liquid Chromatography Tandem Mass Spectrometry; ECL: Electrochemiluminescence; DELFIA: Dissociation-Enhanced Lanthanide Fluorescent Immunoassay, TRFIA : Time-Resolved Fluorescence Immunoassay Analyzer; EIA: Enzyme Immunoassay; ns: not stated

[1]combined model with insulin levels; [2]combined model with risk factors; [3]combined model with BMI

**prior to pregnancy
